# Development of a Rapid Fluorescent Immunochromatographic Test to Detect Respiratory Syncytial Virus

**DOI:** 10.3390/ijms19103013

**Published:** 2018-10-02

**Authors:** Trinh Thi Thuy Tien, Hyun Park, Hien Thi Tuong, Seung-Taek Yu, Du-Young Choi, Seon-Ju Yeo

**Affiliations:** 1 Zoonosis Research Center, Department of Infection Biology, School of Medicine, Wonkwang University, 460, Iksan-daero, Iksan 54538, Korea; trinhthithuytien.k56@hus.edu.vn (T.T.T.T.); hyunpk@wku.ac.kr (H.P.); tuonghien23@gmail.com (H.T.T.); 2 Department of Pediatrics, School of Medicine, Wonkwang University, 460, Iksan-daero, Iksan 54538, Korea; yudoc@wku.ac.kr (S.-T.Y.); cdy8118@wku.ac.kr (D.-Y.C.)

**Keywords:** monoclonal antibody, respiratory syncytial virus, nucleoprotein, fluorescent immunochromatographic test, clinical study

## Abstract

Human respiratory syncytial virus (RSV) is one of the most common viruses infecting the respiratory tracts of infants. The rapid and sensitive detection of RSV is important to minimize the incidence of infection. In this study, novel monoclonal antibodies (mAbs; B11A5 and E8A11) against RSV nucleoprotein (NP) were developed and applied to develop a rapid fluorescent immunochromatographic strip test (FICT), employing europium nanoparticles as the fluorescent material. For the FICT, the limits of detection of the antigen and virus were 1.25 µg/mL and 4.23 × 10^6^ TCID_50_/mL, respectively, corresponding to 4.75 × 10^6^ ± 5.8 ×10^5^ (mean ± SD) RNA copy numbers per reaction mixture for RSV NP. A clinical study revealed a sensitivity of 90% (18/20) and specificity of 98.18% (108/110) for RSV detection when comparing the performance to that of reverse transcription polymerase chain reaction (RT-PCR), representing a 15% improvement in sensitivity over the SD Bioline rapid kit. This newly developed FICT could be a useful tool for the rapid diagnosis of RSV infection.

## 1. Introduction

Worldwide, acute respiratory tract infections during infancy and childhood are mainly caused by human respiratory syncytial virus (RSV) [1], and the most common cause of bronchiolitis and pneumonia among children aged <1 year is nadir concentration of protective maternal immunoglobulin G (IgG) transferred to the fetus [2]. RSV is a widespread human pathogen because the immunity that is obtained after RSV infection might not be protective, frequently leading to reinfection. Approximately 90% of children become infected within the first two years of life, and this virus frequently re-infects older children and adults. The majority of patients with RSV infection develop upper respiratory illness, but a significant minority will present with lower respiratory tract illness, predominantly in the form of bronchiolitis.

The early diagnosis of RSV infection is essential, and the available methods for diagnosis that use respiratory samples include virus culture, molecular diagnostics, and molecular assays. The rapid and sensitive detection of RSV is important to implement infection control measures, thus preventing hospitalizations, as RSV has been recognized as a major risk in pediatric wards [3].

Human RSV has been reclassified to species *Human orthopneumovirus*, belonging to the *Pneumoviridae* family and the genus *Orthopneumovirus* [4]. RSV was discovered in chimpanzees in 1955, and subsequently confirmed to be a human pathogen shortly thereafter. Several animal RSVs in the same genus as human RSV do not infect humans. Its non-segmented, single-stranded, negative-sense RNA genome is 15.2 kb in length and contains 10 genes. In the 3′ to 5′ direction, the genome contains genes for two non-structural proteins (NS1 and NS2), a nucleoprotein (NP), a phosphoprotein (P), a matrix protein (M), a small hydrophobic protein (SH), an attachment glycoprotein (G), a fusion glycoprotein (F), an M2 protein, and a polymerase (L) [5].

To date, many monoclonal antibodies have been developed against the fusion protein of RSV, and the effect of the antibodies on RSV disease has been widely studied [6,7,8,9,10,11]. However, overall, for reported RSV immunoassays, the pooled sensitivity and specificity are 80% (95% confidence interval (CI), 76–83%) and 97% (95% CI, 96–98%), respectively [12]. Polyclonal antibodies that are produced against the human RSV nucleoprotein (NP) have been reported to detect RSV in immunofluorescence assays [13]. In this study, newly developed monoclonal antibodies against NP were used to develop an immunoassay, and the clinical diagnostic performance of this immunoassay was evaluated.

## 2. Results

### 2.1. Development of Monoclonal Antibody

**Figure 1:**
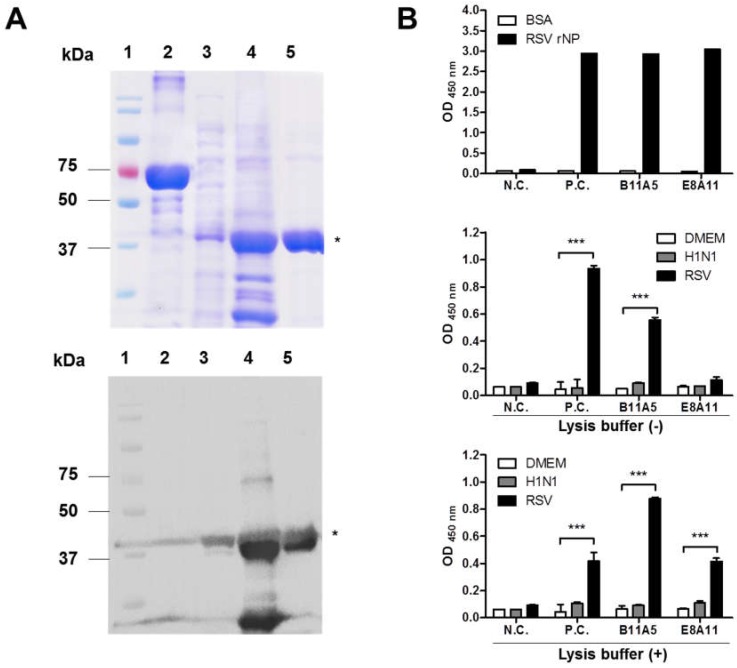
Development of antigen and antibody. (**A**) Recombinant respiratory syncytial virus nucleoprotein (RSV-NP) was expressed in an *E. coli* system and purified using a nickel nitrilotriacetic acid (Ni-NTA Agarose). The upper panel shows the SDS-PAGE results, and the lower presents Western blot results with an anti-His-6× tag antibody. 1, marker; 2, bovine serum albumin (BSA); 3, supernatant after induction; 4, pellet after induction; 5, purified RSV recombinant NP (rNP). The asterisk (*) indicates the target band. (**B**) Secreted antibodies in the supernatants of two hybridomas were tested with recombinant NP (10 µg/mL) using Dulbecco’s Modified Eagle Medium (DMEM) and Influenza A H1N1 virus as negative control. Finally, purified antibodies (B11A5 and E8A11) were tested with virus (1 × 10^7^ TCID_50_/mL) by performing an indirect ELISA in the absence or presence of lysis buffer. Two-way ANOVA. *** *p* < 0.001.

To develop monoclonal antibody (mAb) for detection of the RSV nucleoprotein (NP), we used the full-length amino acid (aa) sequence of NP (391aa GenBank: ALS35585.1) to produce the antigen. The RSV NP gene was cloned into pET21(b+) for expression in an *E. coli* system. The expressed RSV NP antigen was used for further purification, resulting in a dominant band at 46 kDa after SDS-PAGE and Western blot analysis using an anti-His tag antibody (Figure 1A).

Initially, hybridomas were selected based on reactivity by performing an ELISA. Culture supernatants were screened for their ability to detect the recombinant antigen. From this, two hybridomas (B11A5 and E8A11) were produced, and the secreted antibodies from each were purified and tested for RSV virus reactivity by indirect ELISA (Figure 1B). B11A5 reacted with RSV (1 × 10^7^ TCID_50_/mL), but E8A11 significantly bound RSV at the same titer in the presence of lysis buffer (*p* < 0.001). H1N1 virus was not detectable with either mAb in the absence or presence of lysis buffer.

### 2.2. Characterization of Novel Monoclonal Antibodies

**Figure 2:**
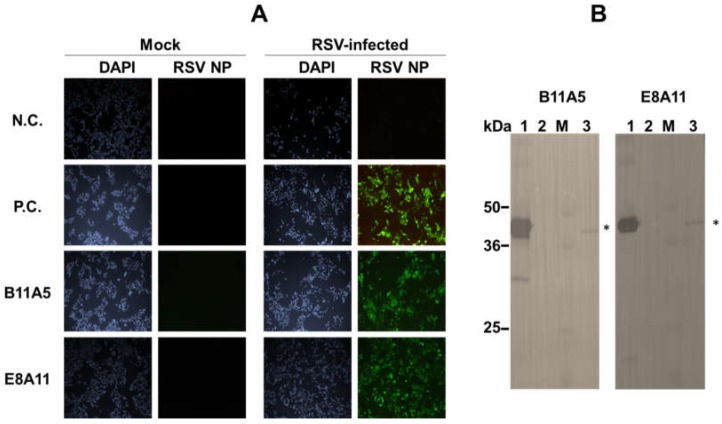
Characterization of monoclonal antibodies against respiratory syncytial virus (RSV). (**A**) Cells were independently infected with the virus for 24 h and fixed with 4% paraformaldehyde. After fixation, cells were treated with lysis buffer and washed with phosphate buffered saline with 0.1% Tween-20 (PBST) three times. Green fluorescence was detected with a fluorescein isothiocyanate (FITC)-conjugated secondary antibody. N.C., negative sera; P.C., commercial anti-RSV NP antibody; Mock, uninfected. All images were acquired by resolution power setting with 100×. (**B**) Western blotting was conducted using an RSV-infected cell pellet. 1, purified RSV recombinant nucleoprotein (rNP; 5 µg/lane); 2, BSA (5 µg/lane); 3, marker; 4, RSV (1 × 10^6^ TCID_50_/mL)-infected cell pellet (4 µg/lane). Asterisk indicates RSV NP protein.

**Figure 3:**
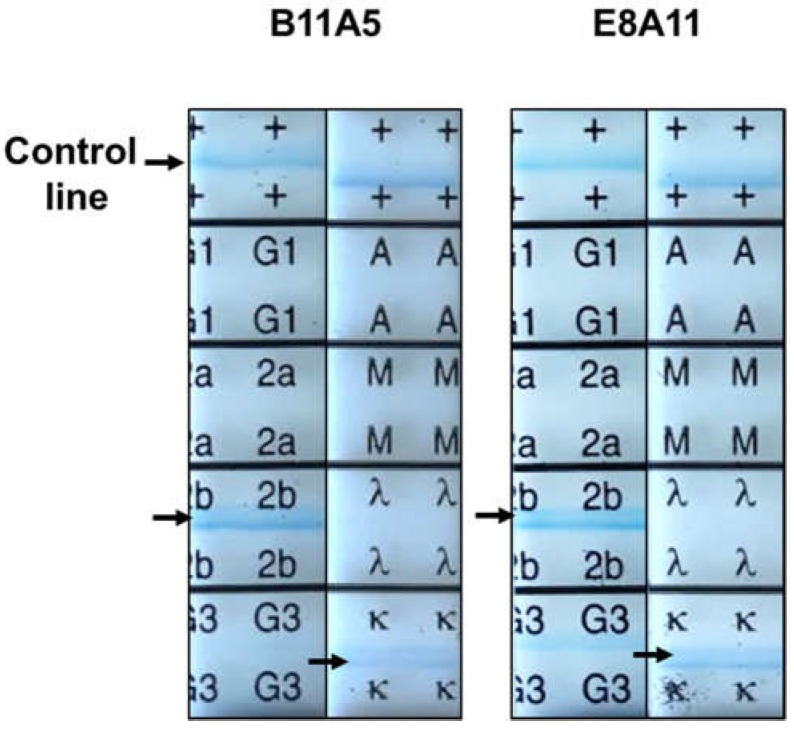
Isotyping of monoclonal antibodies used for the detection of respiratory syncytial virus. IgG subclasses of novel monoclonal antibodies were determined using an Isostrip. Purified antibodies (1 µg/mL) were reacted with the strip for 5 min and read by eye. Black arrows indicate a positive signal.

To further characterize mAbs, viral reactivity was visualized by performing an immunofluorescence assay (IFA). E8A11 was not able to detect RSV in the absence of a suitable lysis buffer, which was confirmed by IFA, as shown Figure S1. After investigation of SDS and pH, lysis buffer (0.1 M tris, 0.1 M ethylenediaminetetraacetic acid (EDTA), 1% triton X-100, and 1% SDS. pH 8.0) was found to be suitable for the detection of virus by the two antibodies using IFA. The reactivity of the mAbs to RSV in the presence of lysis buffer was shown by IFA (Figure 2A). In the presence of lysis buffer, positive signals in RSV-infected cells were observed for both antibodies and a commercial monoclonal antibody against RSV NP. Western blotting revealed a major band at 46 kDa, indicating the reactivity of B11A5 and E8A11 against denatured RSV NP (Figure 2B). The B11A5 and E8A11 isotypes were both found to be IgG2b (Figure 3).

### 2.3. Performance of Fluorescence-Linked Immunosorbent Assay (FLISA)

This study aimed to generate a rapid fluorescent diagnostic system; however, before the antibodies were applied to a rapid diagnostic strip, the performance of fluorescent-conjugated antibodies was evaluated by fluorescence-linked immunosorbent assay (FLISA).

**Figure 4:**
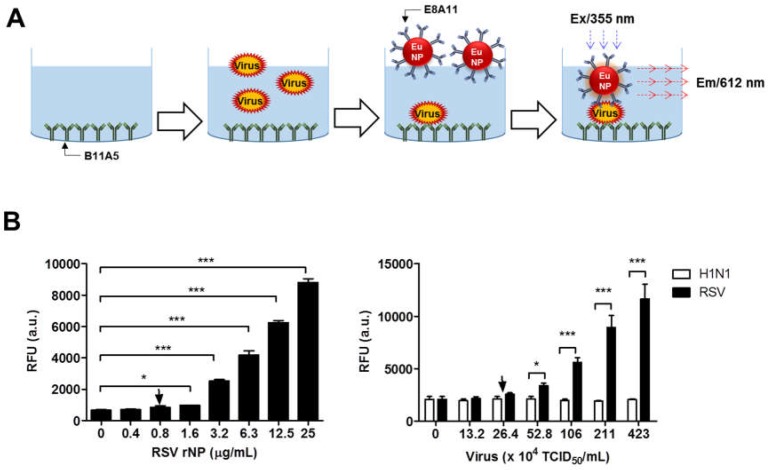
Performance of sandwich fluorescence-linked immunosorbent assay (FLISA) using two novel monoclonal antibodies for the detection of respiratory syncytial virus (RSV). (**A**) Sandwich FLISA using B11A5 (capture) and europium nanoparticle (Eu NP)-conjugated E8A11 (detection) was conducted with serial dilutions of RSV recombinant nucleoprotein (rNP) and virus. Fluorescence was measured for bound Eu NP-conjugated E8A11 (excitation at 355 nm and emission at 612 nm). (**B**) Serially diluted RSV rNP antigen, from 0.4 µg/mL to 25 µg/mL, and virus, from 13.20 × 10^4^ to 4.23 × 10^6^ TCID_50_/mL, were tested by FLISA. H1N1 was used as a negative virus control. Data (*n* = 3) are shown as the mean ± SD. a.u., arbitrary units; LOD, limit of detection. One-way ANOVA; * *p* < 0.05; *** *p* < 0.001.

Figure 4A schematically illustrates the europium nanoparticle (Eu NP) conjugate-based fluorescent immunochromatographic strip test (FICT). The 96-well plate was coated with the anti-RSV NP-specific antibody (B11A5). In the presence of lysis buffer, analytes (antigen and virus) were applied to the wells, and in the presence of Eu NP-conjugated anti-RSV and NP-specific antibody (E8A11), analytes were detected by measuring the fluorescence intensity. To determine the performance of FLISA, serial two-fold dilutions of RSV rNP, from 0.4 to 25 µg/mL, and two-fold dilutions of H1N1 and RSV, from 13.2 × 10^4^ to 423 × 10^4^ TCID_50_/mL, were used to determine the limit of detection (LOD) of the sandwich FLISA based on the limit of the blank (LOB), as described previously [14]. According to the fluorescent value, the LOD of the B11A5 and E8A11 antibody pair-linked ELISAs was 0.8 µg/mL for RSV rNP and 2.64 × 10^5^ TCID_50_/mL for RSV (Figure 4B).

### 2.4. Development of the FICT

As lateral flow-based rapid diagnostic kits are still widely used and convenient, the development of more sensitive and rapid methods is important and valuable. In this study, a typical lateral flow test strip was combined with fluorescent material to increase sensitivity. 

**Figure 5:**
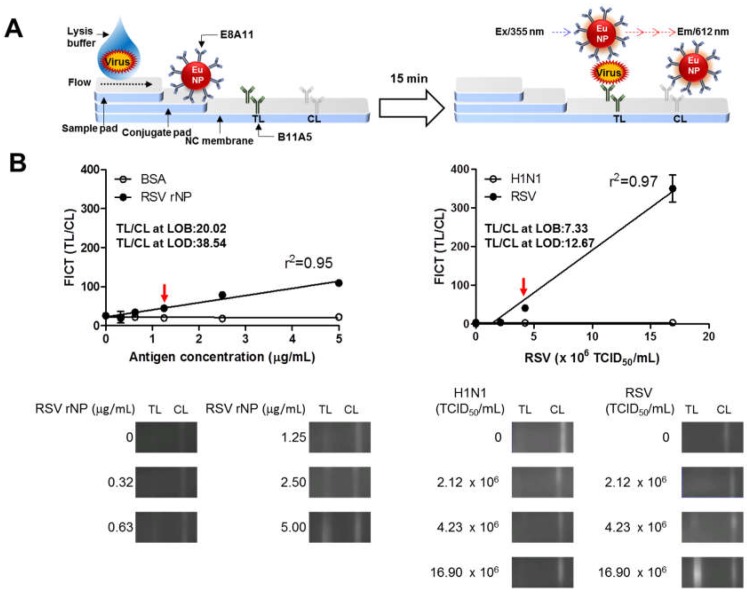
Development of a rapid fluorescence diagnostic system for the detection of respiratory syncytial virus (RSV). (**A**) Schematic diagram of the rapid fluorescence diagnostic system employing a europium nanoparticle (Eu NP)-conjugated RSV-specific antibody. Fluorescence was measured for Eu NPs (excitation at 355 nm and emission at 612 nm). (**B**) Fluorescent immunochromatographic strip test (FICT) employing Eu NP-conjugated antibodies was tested for its limit of detection (LOD) against RSV rNP and RSV. The data (*n* = 3) are shown as the mean ± SD. Linear regression is shown with the line. The red arrow indicates the antigen concentration or virus titer at the LOD. Raw fluorescence images from the test line (TL) and control line (CL) of the FICT are shown in the bottom panel. The signals at the TL and CL were read with a portable strip reader, and the fluorescent values of TL/CL were computed and plotted on the graph.

Figure 5A schematically illustrates the Eu NP conjugate-based FICT. The test strip for the FICT had a conjugate pad for the conjugate and a sample pad for application of the sample on a nitrocellulose membrane. B11A5 and anti-mouse IgG were coated on the test line (TL) and the control line (CL), respectively. To perform the diagnostic assay, the conjugates of E811 and Eu NP were loaded onto the conjugation pad in advance. In the presence of lysis buffer, the conjugate was captured on the TL by analytes. At the CL, anti-mouse IgG recognized the antibody on the conjugate. Fluorescence intensity was digitalized with a portable strip reader in 15 min. Figure 5B indicates the LOD of the RSV rNP and virus. The FICT displayed RSV rNP reactivity with a good correlation (*r*^2^ = 0.95), between 0.3–5 µg/mL, and showed excellent correlation with virus titers (*r*^2^ = 0.97), from 2.12 × 10^6^ to 16.90 × 10^6^ TCID_50_/mL. According to a previous description [14], the LOD of the FICT was 1.25 µg/mL for RSV rNP and 4.23 × 10^6^ TCID_50_/mL for virus. H1N1 virus did not react with the FICT, even at a high titer. The raw data from the FICT are provided in Figures S2 and S3.

### 2.5. Quantitative Real-Time Reverse Transcription Polymerase Chain Reaction (qRT-PCR)

**Figure 6:**
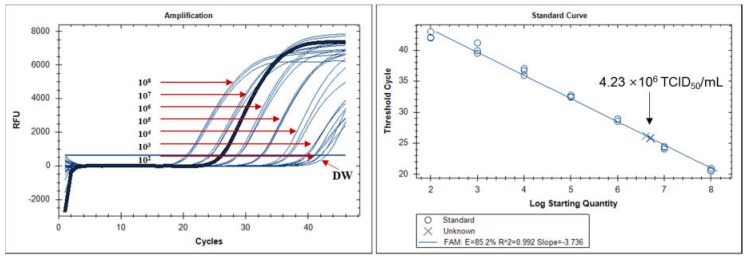
Assessment of fluorescent immunochromatographic strip test (FICT) performance by qRT-PCR. The linear relationship between the cycle threshold (Ct) and RNA copy number of respiratory syncytial virus (RSV) nucleoprotein (NP) was used to produce a standard curve (left panel). The right panel indicates the Ct value (Y-axis) and the RNA copy numbers (X-axis) at a virus titer (4.23 × 10^6^ TCID_50_/mL) corresponding to the limit of detection of the FICT.

To compare the performance of FICT to a molecular diagnostic method, the LOD of FICT was analyzed based on RNA copy number by qRT-PCR. The RNA copy number at the LOD (4.23 × 10^6^ TCID_50_/mL) of the FICT was determined by qRT-PCR. After preparing a virus at 4.23 × 10^6^ TCID_50_/mL, 75 μL of the virus sample was used for RNA extraction. A calibration curve was generated by serially diluting the RNA standard of the RSV NP. A standard curve was created to show the starting copy number of the standard RNA on the X-axis versus the cycle threshold (Ct) on the Y-axis. The plot of the standard curve of Ct values against the logarithmic dilutions produced an *r*^2^ value = 0.992, and the slope (−3.736) corresponded to an efficiency in the range of 85.2% for RSV NP, which was close to that of the optimized protocol. The LOD of the FICT corresponded to a Ct value of 25.95 ± 0.28 (mean ± SD) and an RNA copy number/reaction mixture of 4.75 × 10^6^ ± 5.8 × 10^5^ (mean ± SD) for RSV NP (Figure 6).

### 2.6. Clinical Performance of the FICT

To evaluate the performance of the FICT to diagnose clinical samples, nasopharyngeal swab specimens from patients with confirmed RSV infections, stored at Wonkwang University hospital between 2016 and 2017, were tested. As RSV-positive patients, 10 RSV A-positive and 10 RSV B-positive specimens were tested. The mean age of the RSV-positive patents (eight females and twelve males) was 11.8 months (range, one month to 38 months), and all of the patients were admitted to the hospital (Table 1). Sample collection was performed within 5 d of the onset of illness. A total of 20 patients were positive according to PCR for RSV, and four were negative for RSV according to cell culture. Specifically, the PCR data revealed 100% (20/20; 95% CI: 83.16–100.00%) and 100% 110/110; 95% CI: 96.70–100.00%), respectively. According to the PCR results, six patients showed a positive result for other viruses such as adenovirus (AD), influenza B virus, rhinovirus (HRV), or coronavirus (CoV NL63), indicating potential co-infection with RSV. The average of the cycle threshold (Ct) of PCR is 29.3 and 25.0 for RSV A and RSV B patients, respectively.

As an RSV-negative control group, patients with other diseases, including an unknown disease (*n* = 33), human metapneumovirus (HMPV) (*n* = 28), parainfluenza virus (PIV) (*n* = 40), AD (*n* = 4), human enterovirus (HEV) (*n* = 4), human HRV (*n* = 14), and human bocavirus (HBoV 1/2/3/4) (*n* = 2) were tested. Some of the patients were co-infected with two or three different viruses. The mean age of the RSV-negative patents (56 females and 54 males) was 2.6 years (range, one month to 17 years), and all of the patients were admitted to the hospital. Sample collection was performed within five days of the onset of the illness. The average of Ct values was 27.4 and 29.1.for PIV and HMPV patients, respectively.

**Figure 7:**
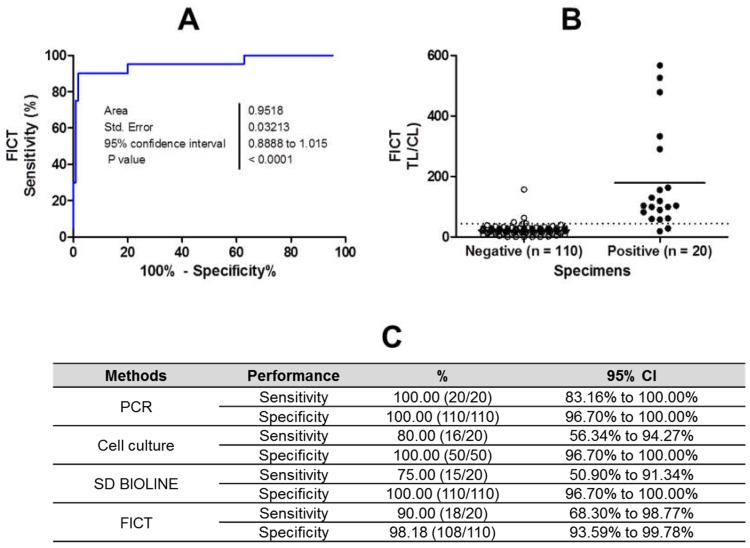
Clinical validation of the fluorescent immunochromatographic strip test FICT with patients infected with respiratory syncytial virus (RSV). The FICT was performed with human specimens collected from patients infected with RSV (*n* = 10) and patients with other diseases (*n* = 50). (**A**) The cut-off value was determined based on the receiver operating characteristic (ROC) curve, representing the sensitivity, specificity, and 95% confidence interval (CI). (**B**) Based on ROC curve analysis, 44.21 was determined as the cut-off value to differentiate RSV infection in a clinical study. This cut-off value for the detection of RSV was applied to determine whether each sample was positive or negative for the presence of the virus, and is indicated in the graph as a dotted line. Nine RSV infection cases showed test line/control line (TL/CL) values that were higher than the threshold value. The non-RSV-infected control group (*n* = 50) had no false-positive cases. (**C**) The sensitivity, specificity, and 95% confidence interval (CI) of the assays were computed for the diagnosis of RSV infection.

For binary diagnostic decisions, the TL/CL threshold cut-off value for RSV was determined to be 53.15 based on receiver operating characteristic (ROC) curve analysis after plotting all of the data using GraphPad Prism; this value was used in the app (positive if TL/CL >53.15, negative otherwise). According to ROC curve analysis, this cut-off value resulted in the highest clinical sensitivity and specificity to diagnose RSV infection (Figure 7A). The ROC curve analysis resulted in an area under the curve (AUC) value of 0.96 (95% CI: 0.889–1.015) for patients (Figure 7B; *p* < 0.0001).

The sensitivity and specificity of the FICT were 90% (18/20; 95% CI: 68.30–98.77%) and 98.18% (108/110; 95% CI: 93.59–99.78%), respectively, whereas the rapid diagnostic test (RDT) SD kit (SD Bioline) produced values of 75% (15/20) for sensitivity (95% CI: 50.90–91.34%) and 100% for specificity (110/110; 95% CI: 96.70–100.00%), indicating that the FICT exhibited higher performance compared to that of the RDT for predicting RSV infection. In FICT, two patients of PIV showed false positive values. Three specimens were negative according to the cell culture, and the other was AD-positive (not RSV-positive), resulting in values of 80% for sensitivity (16/20; 95% CI: 56.34–94.27%) and 100% for specificity (100/100; 95% CI: 96.70–100.00%), respectively (Figure 7C). The raw data from the FICT are provided in Figure S4. The sensitivity of the FICT assay was compared to those of PCR and SD RDT for RSV. To evaluate the correlation between PCR and the FICT assay, the kappa statistic was calculated as previously described [15]. FICT (kappa; 0.98) showed better correlation to PCR than RDT (kappa; 0.96) (Table 2).

## 3. Discussion

RSV is the most common viral cause of pediatric bronchiolitis and pneumonia worldwide in infants <6 months of age [16,17]. However, the clinical manifestations of RSV are indistinguishable from other etiologies of acute respiratory infection [18]. The performance of current clinical RSV rapid diagnostic kits is approximately 87.5–93% sensitivity and 86–96% specificity [19,20]. Due to this low sensitivity, alternative methods with improved biophysical approaches for RSV detection, and employing easy-to-perform and rapid diagnostic systems, are needed.

The current detection methods for RSV involve fluorescent material or plasmon, which improve immunoassay sensitivity [21,22]. Due to its speed, convenience, low cost, portability, and ability to provide quantifiable results, the application of fluorescent material could lead to promising point-of-care diagnostic tools to screen patients with suspected respiratory infection or other types of infectious diseases. One of the most promising fluorescent materials is europium nanoparticles; although it has been used for influenza, it has not been used for RSV diagnostic systems. 

To develop an effective detection method, a suitable target for making antibodies needs to be identified. Fusion proteins have been commonly used for making antibodies to neutralize RSV [23]. However, despite being a surface antigen, a fusion protein has limitations as a diagnostic target based on the cross-reactivity of paramyxovirus with monoclonal antibodies [24]. In this study, we searched for highly conserved proteins, identifying NP. There have been some reports on the use of NP for diagnostic assays to detect RSV, although the sensitivity was not satisfactory [20,25]. In most cases, the sensitivity was below 70%, and the LOD was not reported.

Recently, the DNA aptamers for detecting the RSV G protein were reported to have an LOD of 8.5 × 10^5^ PFU/mL [26]. Upon converting this value to 5.95 × 10^5^ TCID_50_/mL, using the formula PFU (mL)/TCID_50_ (mL) = 0.7 [27], a higher performance than our FICT (with a LOD of 4.23 × 10^6^ TCID_50_/mL) was indicated. However, this study tested a spiked virus in specimens rather than clinical patient samples.

The sensitivity/specificity of the SD RSV kit was reported to be 61.3%/100%, as compared to RT-PCR [28]; the sensitivity of the RDT SD RSV that was developed in our study was 75%/100%. We consider that the sensitivity of our FICT might be 15% higher than that of the commercial SD RSV RDT, as our assay had a 90% sensitivity for PCR-positive patients. The range of viral loads for respiratory specimens was 3.2 × 10^3^–1.5 × 10^7^ RNA copies per mL [29], and thus, our FICT corresponds to a RNA copy number/reaction mixture value of 4.75 × 10^6^ ± 5.8 × 10^5^ (mean ± SD) for RSV NP, and the established LOD might be useful to diagnose RSV infection in patients. FICT showed that it could detect RSV in the co-infected specimens more accurately than SD RDT, with a higher positive rate (6/6) for FICT than for SD RDT (4/6). However, FICT was not able to detect RSVB in one patient (P45.11), although the cell culture for this patient was positive and the patient possessed a high amount of RSV RNA (Ct value of 22). SD RDT was also negative for this patient (P45.11). Except for this patient, FICT was able to detect RSV in all of the patients with Ct values of 17–25. However, SD RDT did not detect five patients with Ct values of 22–35. Although PCR showed a high performance in diagnosing RSV, it is still limited by its complexity and high cost [30]. Furthermore, upper respiratory infection is common in the winter months, indicating that acute and prior infection with these pathogens cannot be distinguished by PCR [31].

Therefore, simple, rapid antigen-detection tests offer potential advantages that are associated with point-of-care testing (POCT) over PCR, and our FICT method can improve the performance of POCT. 

Fusion antigen-based POCT (QuickVue^®^ RSV Test Kit) demonstrated a sensitivity of 90% and specificity of 98.8% [32]. In this study, our lysis buffer was indispensable for FICT because the clinical performance of FICT was comparable with that of a fusion antigen-based QuickVue^®^ RSV kit. The lysis buffer containing detergent (1% SDS) was efficient for the antibody to detect RSV.

The limitation of this study was the small patient population that was used to evaluate the clinical performance of the FICT. Thus, further studies are needed to assess the accuracy of the FICT.

## 4. Materials and Methods

### 4.1. Reagents

Europium nanoparticles (200-nm diameter) were purchased from Bangs Laboratories Inc. (Fishers, IN, USA). Aliphatic amine latex beads (100-nm diameter) were purchased from Life Technology (Carlsbad, CA, USA). *N*-(3-Dimethylaminopropyl)-*N*′-ethylcarbodiimide hydrochloride (EDC) and *N*-hydroxysulfosuccinimide sodium salt (Sulfo-NHS) were purchased from Thermo Fisher Scientific (Waltham, MA, USA). Anti-RSV Fusion and anti-RSV nucleoprotein (NP) were purchased from Abcam (Cambridge, UK). Rabbit anti-mouse IgG H&L (horseradish peroxidase (HRP)) and goat anti-mouse IgG H&L (FICT) ab6758 were obtained from Abcam.

### 4.2. Viruses

RSV A (strain KUMC-41) and influenza A virus H1N1 (strain KUMC-76) were obtained from the Korea National Research Resource Center.

### 4.3. Cell Culture and RSV Infection

Hep-2 cells were cultured in Dulbecco’s modified Eagle’s medium (DMEM) supplemented with 10% fetal bovine serum and 1% antibiotic–antimycotic (Invitrogen, Carlsbad, CA, United States) at 37 °C with 5% CO_2_ in a humidified incubator (Sanyo, Osaka, Japan). When monolayers were 80% confluent, cells were infected with RSV at a multiplicity of infection (m.o.i.) of one, which was followed by 120 min of incubation. Then, the supernatant was removed, and complete media were added to the cells, which was followed by incubation at 37 °C for five days. The TCID_50_ assay was conducted as previously reported [33].

### 4.4. Expression of RSV Recombinant Nucleoprotein Antigen

The full-length gene encoding RSV-A NP (GenBank: KT992094.1) was amplified by PCR with two pairs of primers, including the forward primer 5′-GGA TCC GAT GGC TCT TAG CAA AGT C-3′ and the reverse primer 5′-CTC GAG CAT AGG TTG TTC CCT TCA A-3′. The RSV-NP DNA fragment was sub-cloned into pET21b (+) and RSV rNP antigen expression was induced by 0.5 mM of isopropyl β-d-1-thiogalactopyranoside. Total proteins were harvested and purified through Ni-NTA (Thermo Fisher Scientific).

The expression of antigen was confirmed by sodium dodecyl sulfate-polyacrylamide gel electrophoresis (SDS-PAGE) using an anti-mouse 6× his-tag antibody (Thermo Fisher Scientific) diluted 1:10,000. The membrane was then washed three times and incubated with the secondary anti-mouse antibody (anti-mouse IgG conjugated with horseradish peroxidase; Abcam) diluted 1:40,000 in blocking buffer for 30 min at room temperature. After washing five times, the protein bands were visualized by Bio-Rad ChemiDoc XRS+ (Bio-Rad) (Hercules, CA, USA).

### 4.5. Production of Monoclonal Antibody Targeting RSV NP

RSV recombinant nucleoprotein (rNP) (50 µg/100 μL) was mixed with an equal volume of Freund’s complete adjuvant (Sigma-Aldrich, St. Louis, MO, USA) and injected intraperitoneally into six-week-old female BALB/c mice, which were obtained from Orient (Seongnam, Gyeonggi, Korea). Mice were biweekly boosted with RSV rNP (25 μg/100 μL) mixed with an equal volume of Freund’s incomplete adjuvant. The cell fusion technique and indirect ELISA were performed according to previously established protocols [34]. The isotyping of mAbs was performed with Immuno-Type™ mouse mAb isotyping kit (Sigma-Aldrich) following the manufacturer’s instructions.

### 4.6. Immunofluorescence Assay

IFA was performed as described previously [34,35]. Briefly, HEP-2 cells infected with RSV at an m.o.i. of one for 24 h were fixed and incubated with lysis buffer including 0.1 M of tris, 0.1 M of EDTA, 1% triton, and 1% SDS (pH 8.0) for 20 min. Cells were blocked with 5% bovine serum albumin (BSA) in PBS-T at room temperature for 2 h. After stringent washing, the coverslips were incubated with 1 µg/well anti-RSV NP for 2 h at room temperature, and then incubated with the fluorescein isothiocyanate (FITC)-conjugated goat anti-mouse IgG H&L for 1 h. Finally, the coverslips were dried and mounted with mounting medium containing 4′,6-diamidino-2-phenylindole (DAPI) (Vector lab, Burlingame, CA, USA). Fluorescence microscopic images were acquired using a fluorescence microscope (Olympus, Tokyo, Japan) at 400× magnification.

### 4.7. Conjugation of Europium Nanoparticles

Antibodies were covalently conjugated to europium nanoparticles as previously published [34]. Briefly, 0.13 mM of EDC and 10 mM of Sulfo-NHS were added to a mixture of 500 µL of 0.1 M tris-HCl (pH 7.0) and 10 µL of Eu NPs and incubated for 1 h at 25 °C. The activated Eu NPs were mixed with 45 µL of Ab (E8A11) in 500 µL of 0.1 M NaH_2_PO_4_ (pH 8.0) and allowed to react for 2 h at 30 °C. After centrifugation at 27,237× *g* for 5 min, the Eu NP-conjugated antibodies were collected, washed with 2 mM of PBS (pH 8.0), re-suspended in 200 µL of storage buffer (1% BSA in PBS), and stored at 4 °C.

### 4.8. Sandwich Fluorescent-Linked Immunosorbent Assay

Black 96-well plates were coated with B11A5 (10 µg/mL) at 4 °C overnight. After washing three times with PBS-T, analytes (antigen or virus) were applied to each well in the presence of 100 µL of lysis buffer for 1 h at 37 °C. Unbound analytes were removed with stringent washing, and 100 µL of Eu-NP-E8A11 conjugate (150 nM antibodies) was applied to the plates, which was followed by incubation at 37 °C for 1 h. Fluorescence was measured using an Infinite F200 microplate reader system (TECAN, Männedorf, Switzerland; excitation 355 nm/emission 612 nm).

### 4.9. Lateral Flow Test Assay for Fluorescent Immunochromatographic Test

FICT was conducted as described previously [34]. The TL of the strip was prepared by coating it with 1 mg/mL of B11A5 and the CL was prepared by coating it with 0.5 mg/mL of polyclonal goat anti-mouse IgG. To perform FICT, 6 µL of Eu NP-conjugated E8A11 Ab (7.5 nM) was dropped onto the conjugate pad, and a mixture of 75 µL of sample and 75 µL of lysis buffer (100 mM of tris-HCl, pH 8.0, 100 M of EDTA, 1% SDS, and 1% triton X-100) was dropped onto the sample pad, and lateral flow assay was conducted for 15 min. The test strip results were read with a portable fluorescent strip reader at excitation and emission wavelengths of at 355 nm and 612 nm, respectively (Medisensor, Daegu, Korea) [36].

### 4.10. RSV Antigen Immunochromatographic Assay Test

The RSV antigen test (SD bioline, Abbott, Santa Clara, CA, USA) was performed using 200 μL of nasopharyngeal specimen mix with the same volume of the provided reagent; then, the RSV strip test was added to this mixture. After lateral flow for 15 min, the results were read by the naked eye.

### 4.11. Real-Time RT-PCR

To evaluate the performance of the immunoassay, RSV qRT-PCR was used for the NP gene as a reference assay. Primers and probes targeting the gene encoding the RSV NP were prepared as previously described [37]. To determine the Ct values corresponding to the LOD of the FICT, qRT-PCR was performed using a Quantitect Probe RT-PCR Kit (QIAGEN, Hilden, Germany) and a CFX96 Real-Time PCR Detection System. To produce a standard for the determination of the RNA copy number, the template was cloned into pGEM-T Easy (Promega, Madison, WI, USA). In vitro transcription reactions were performed using a RiboMax T7 transcription kit (Promega).

### 4.12. Ethical Considerations

This study was approved by the Wonkwang University Hospital Institutional Review Board (Approval No. WKIRB-201603-BR-015) which was approved in 15 July 2016. All of the patients agreed to participate in the study, and informed consent was obtained before obtaining specimens. All of the experiments and methods were performed in accordance with relevant guidelines and regulations. Nucleic acid was extracted from specimens using the RNeasy Mini kit (Qiagen, Mississauga, ON, Canada) and amplified by commercial multiplex assay (Seegene Inc., Seoul, Korea). The R-Mix rapid cell culture method (Quidel, San Diego, CA, USA) was used for diagnoses of specimens.

### 4.13. Statistics

In general, all of the data are provided as the mean ± standard deviation (SD) of biological replicates, and were plotted using GraphPad Prism 5.0 (GraphPad, La Jolla, CA, USA).

## 5. Conclusions

FICT improves the sensitivity of conventional RDT to diagnose RSV infection. This assay could help promptly identify patients infected with RSV so that necessary treatments can be started immediately after diagnosis.

## Figures and Tables

**Table 1:** Characteristics of clinical specimens.

Specimen		Collection of Sample (mm-dd-yyyy)	Age	Sex	PCR (Cycle Threshold)	Culture	SD RDT ^a^	FICT	
								TL/CL ^b^	Binary Decision
RSV-positive cases	P40.1	10/23/2017	1 y 8 m	M	AD (26), RSV B (25), HRV (31)	Positive	Positive	99.37	Positive
	P43.6	11/14/2017	0 y 7 m	M	RSV B (19)	Positive	Positive	566.96	Positive
	P43.7	11/15/2017	2 y 5 m	M	RSV B (26)	Positive	Positive	129.65	Positive
	P44.1	11/20/2017	2 y 0m	M	RSV B (33)	AD	Positive	57.41	Positive
	P45.11	11/30/2017	0 y 4 m	F	RSV B (22)	Positive	Negative	18.75	Negative
	P49.26	01/09/2018	0 y 9 m	M	Flu B (39), RSV B (17)	Positive	Positive	525.56	Positive
	P50.3	01/15/2018	1 y 6 m	F	RSV B (24)	Positive	Positive	155.33	Positive
	P50.14	01/18/2018	0 y 2 m	M	RSV B (25)	Positive	Positive	478.19	Positive
	P52.1	01/29/2018	1 y 4 m	F	HRV (21), RSV B (35), CoV (28)	Negative	Negative	162.89	Positive
	P53.7	02/09/2018	0 y 1 m	M	RSV B (24)	Positive	Negative	103.32	Positive
	P1.5	10/26/2016	0 y 5 m	F	RSV A (31)	Positive	Positive	61.42	Positive
	P6.1	11/28/2016	0 y 1 m	M	RSV A (33)	Positive	Positive	89.4	Positive
	P6.3	11/28/2016	2 y 8 m	M	RSV A (29)	Positive	Positive	59.32	Positive
	P8.2	12/12/2016	3 y 2 m	F	AD (35), PIV-1 (32), RSV A (25)	Negative	Negative	99.28	Positive
RSV-positive cases	P8.3	12/12/2016	0 y 9 m	F	AD (36), RSV A (31)	Negative	Positive	103.21	Positive
	P8.6	12/14/2016	0 y 4 m	F	HRV (32), RSV A (29)	Positive	Positive	118.5	Positive
	P10.2	12/26/2016	0 y 1 m	M	RSV A (23)	Positive	Positive	290.37	Positive
	P11.7	1/4/2017	0 y 7m	M	RSV A (35)	Positive	Negative	28.44	Negative
	P12.3	1/9/2017	0 y 1m	F	RSV A (33)	Positive	Positive	81.57	Positive
	P13.2	1/18/2017	0 y 9m	M	RSV A (22)	Positive	Positive	332.91	Positive
RSV-negative cases	P23.4	03/31/2017	0 y 10 m	F	PIV-3 (25)	PIV	Negative	10.6	Negative
	P26.1	05/08/2017	0 y 4 m	M	Negative	PIV	Negative	12.97	Negative
	P26.2	05/15/2017	0 y 11 m	F	AD (23)	Negative	Negative	27.01	Negative
	P26.5	05/17/2017	13 y 6 m	M	PIV-3 (17)	PIV	Negative	19.78	Negative
	P27.2	05/25/2017	5 y 9 m	F	Negative	Negative	Negative	14.6	Negative
	P27.4	05/30/2017	1 y 1 m	M	Negative	Negative	Negative	10.51	Negative
	P28.3	06/14/2017	0 y 3 m	F	AD (19), HBoV 1/2/3/4 (28)	AD	Negative	17.11	Negative
	P29.1	06/19/2017	0 y 5 m	M	Negative	Negative	Negative	21.16	Negative
	P29.2	06/26/2017	12 y 10 m	F	Negative	Negative	Negative	24.66	Negative
	P29.3	06/26/2017	15 y 6 m	F	Negative	Negative	Negative	25.48	Negative
	P29.4	06/27/2017	2 y 2 m	M	Negative	Negative	Negative	20.43	Negative
	P30.1	07/03/2017	0 y 10 m	M	PIV-4 (29)	Negative	Negative	9.54	Negative
	P30.2	07/03/2017	1 y 0 m	M	Negative	Negative	Negative	25.12	Negative
	P30.3	07/03/2017	0 y 2 m	M	Negative	Negative	Negative	21.22	Negative
	P30.4	07/03/2017	0 y 1 m	F	Negative	Negative	Negative	21.73	Negative
	P30.5	07/04/2017	1 y 0 m	F	Negative	PIV,	Negative	24.03	Negative
	P30.7	07/04/2017	3 y 11 m	M	Negative	Negative	Negative	17.85	Negative
	P30.8	07/05/2017	2 y 6 m	F	PIV-4 (16), HEV (23)	Negative	Negative	18.66	Negative
	P30.11	07/06/2017	0 y 1 m	F	HEV (32)	Negative	Negative	0	Negative
	P30.12	07/07/2017	1 y 6 m	F	Negative	Negative	Negative	31	Negative
	P30.13	07/08/2017	6 y 7 m	F	HEV (29)	Negative	Negative	0	Negative
	P30.14	07/10/2017	1 y 10 m	F	Negative	Negative	Negative	0	Negative
	P30.16	07/11/2017	5 y 8 m	M	Negative	Negative	Negative	30.95	Negative
	P30.17	07/11/2017	0 y 2 m	M	PIV-4 (26)	Negative	Negative	21.32	Negative
	P30.19	07/12/2017	0 y 1 m	F	PIV-4 (32)	Negative	Negative	0	Negative
	P30.20	07/12/2017	2 y 8 m	M	HRV (33), HEV (31)	Negative	Negative	20.5	Negative
	P41.1	10/30/2017	4 y 0 m	F	PIV-1 (22)	PIV	Negative	7.63	Negative
	P41.4	1/01/2017	1 y 7 m	M	Negative	PIV	Negative	6.5	Negative
	P41.5	11/02/2017	7 y 5 m	F	Negative	Negative	Negative	26.85	Negative
	P41.6	11/02/2017	4 y 7 m	M	Negative	Negative	Negative	29.7	Negative
	P43.3	11/13/2017	1 y 2 m	F	Negative	Negative	Negative	21.99	Negative
	P43.8	11/15/2017	1 y 7 m	F	AD (31), HRV (35)	Negative	Negative	24.06	Negative
	P43.9	11/15/2017	1 y 2 m	F	Negative	Negative	Negative	15.95	Negative
	P43.10	11/15/2017	5 y 10 m	M	Negative	Negative	Negative	24.23	Negative
	P44.2	11/20/2017	5 y 11 m	M	Negative	Negative	Negative	22.29	Negative
	P44.3	11/20/2017	1 y 6 m	F	Negative	Negative	Negative	21.24	Negative
	P44.6	11/22/2017	1 y 10 m	M	Negative	Negative	Negative	23.65	Negative
	P44.8	11/23/2017	1 y 4 m	M	PIV-1 (19)	PIV	Negative	28.39	Negative
	P44.9	11/24/2017	15 y 2 m	M	HRV (28)	Negative	Negative	26.51	Negative
	P45.1	11/27/2017	17 y 3 m	F	Negative	Negative	Negative	23.3	Negative
	P45.3	11/27/2017	17 y 7 m	M	Negative	Negative	Negative	15.85	Negative
	P45.4	11/27/2017	3 y 1 m	M	Negative	Negative	Negative	10.51	Negative
	P45.6	11/28/2017	2 y 5 m	M	HRV (35)	Negative	Negative	20.74	Negative
RSV-negative cases	P45.8	11/29/2017	10 y 3 m	M	HRV (38)	Negative	Negative	28.84	Negative
	P45.10	11/29/2017	4 y 0 m	F	HRV (37)	Negative	Negative	24.3	Negative
	P46.1	12/04/2017	6 y 2 m	M	Negative	Negative	Negative	13.75	Negative
	P47.1	12/18/2017	7 y 0 m	M	Negative	Negative	Negative	24.32	Negative
	P47.8	12/17/2017	0 y 3 m	M	HRV (29)	Negative	Negative	13.61	Negative
	P47.12	12/16/2017	3 y 0 m	M	AD (30)	Negative	Negative	20.12	Negative
	P47.15	12/19/2017	2 y 11 m	F	Negative	Negative	Negative	14.84	Negative
	P19.3	3/3/2017	1 y 1 m	F	Negative	PIV-3	Negative	23.79	Negative
	P23.6	4/3/2017	0 y 4 m	F	PIV-4 (32)	Negative	Negative	15.86	Negative
	P24.14	4/18/2017	0 y 3 m	M	HRV (30)	PIV-3	Negative	26.75	Negative
	P24.20	4/20/2017	1 y 9 m	M	Negative	PIV-3	Negative	18.87	Negative
	P25.6	4/30/2017	3 y 6 m	M	Negative	PIV-3	Negative	1.95	Negative
	P25.7	5/2/2017	1 y 5 m	M	PIV-3 (23)	PIV-3	Negative	29.74	Negative
	P34.2	8/28/2017	4 y 7 m	F	PIV-4 (38)	Negative	Negative	9.86	Negative
	P40.11	10/27/2017	1 y 6 m	M	PIV-1 (34)	Negative	Negative	3.89	Negative
	P47.24	12/26/2017	1 y 0 m	F	PIV-1 (33)	Negative	Negative	18.7	Negative
	P47.35	12/27/2017	7 y 2 m	F	PIV-1 (21)	PIV-1	Negative	30	Negative
	P49.34	1/11/2018	2 y 8 m	M	PIV-1 (19)	PIV-1	Negative	0	Negative
	P55.10	2/23/2017	1 y 7 m	F	PIV-1 (34)	Negative	Negative	32.67	Negative
	P62.6	4/11/2018	2 y 2 m	F	PIV-3 (25)	PIV-3	Negative	13.56	Negative
	P62.8	4/12/2018	2 y 9 m	F	PIV-1 (21)	PIV-1	Negative	38.52	Negative
	P63.10	4/19/2018	0 y 11 m	M	PIV-3 (19)	PIV-3	Negative	22.4	Negative
	P63.16	4/21/2018	0 y 4 m	F	PIV-3 (36)	Negative	Negative	28.63	Negative
	P63.17	4/21/2018	2 y 2 m	F	PIV-3 (30)	PIV-3	Negative	63.31	Negative
	P64.3	4/23/2018	0 y 3 m	F	PIV-3 (24)	PIV-3	Negative	28.55	Negative
	P64.4	4/24/2018	2 y 1 m	F	PIV-3 (23)	PIV-3	Negative	9.22	Negative
	P65.1	4/30/2018	0 y 5 m	M	PIV-3 (35)	Negative	Negative	14.92	Negative
	P65.2	4/30/2018	0 y 4 m	M	PIV-3 (38)	Negative	Negative	17.82	Negative
	P65.8	5/2/2018	2 y 3 m	M	PIV-3 (28)	PIV-3	Negative	24.15	Negative
	P65.16	5/4/2018	2 y 2 m	F	PIV-3 (17)	PIV-3	Negative	18.61	Negative
	P65.17	5/4/2018	2 y 8 m	F	PIV-1 (31)	PIV-1	Negative	156.92	Negative
	P66.9	5/11/2018	2 y 1 m	F	PIV-3 (23)	PIV-3	Negative	14.37	Negative
	P66.10	5/11/2018	0 y 10 m	M	PIV-3 (22)	PIV-3	Negative	4.52	Negative
	P67.4	5/15/2018	1 y 4 m	F	PIV-3 (27)	PIV-3	Negative	42.32	Negative
	P67.6	5/15/2018	1 y 8 m	F	PIV-3 (28)	PIV-3	Negative	20.06	Negative
	P67.8	5/16/2018	0 y 3 m	F	PIV-3 (21)	PIV-3	Negative	12.62	Negative
	P67.9	5/16/2018	2 y 2 m	F	PIV-3 (32)	Negative	Negative	20.88	Negative
	P68.1	5/21/2018	0 y 3 m	F	PIV-3 (20)	PIV-3	Negative	13.14	Negative
	P68.4	5/23/2018	0 y 2 m	F	PIV-3 (34)	Negative	Negative	28.37	Negative
	P20.3	3/7/2017	0 y 4 m	M	HMPV (32)	Negative	Negative	23.44	Negative
	P20.7	3/10/2017	0 y 7 m	F	HMPV (20)	HMPV	Negative	24.39	Negative
	P21.6	3/14/2017	1 y 8 m	M	HRV (39), HMPV (34)	Negative	Negative	29.22	Negative
	P21.11	3/17/2017	0 y 9 m	M	HMPV (37)	Negative	Negative	35.52	Negative
	P21.13	3/17/2017	2 y 4 m	F	HMPV (34)	Negative	Negative	7.64	Negative
	P21.14	3/20/2017	1 y 11 m	M	HRV (32), PIV-4 (37), HMPV (29)	Negative	Negative	17.96	Negative
	P22.1	3/21/2017	2 y 8 m	M	HMPV (39)	Negative	Negative	34.92	Negative
	P22.5	3/23/2017	0 y 7 m	F	HRV (29), HMPV (34)	Negative	Negative	25.35	Negative
	P23.7	4/3/2017	13 y 8 m	M	HMPV (30)	Negative	Negative	21.46	Negative
	P23.10	4/4/2017	0 y 10 m	M	HMPV (21)	HMPV	Negative	22.34	Negative
	P23.20	4/7/2017	4 y 5 m	M	HMPV (23)	HMPV	Negative	24.53	Negative
	P23.21	4/7/2017	6 y 10 m	F	HMPV (39)	Negative	Negative	9.74	Negative
	P24.17	4/19/2017	0 y 5 m	F	HMPV (35)	Negative	Negative	21.93	Negative
	P25.5	4/28/2017	7 y 5 m	F	HMPV (31)	Negative	Negative	23.82	Negative
	P55.8	2/22/2018	0 y 5 m	M	HMPV (24)	HMPV	Negative	27.87	Negative
	P62.2	4/10/2018	1 y 0 m	M	HMPV (34)	Negative	Negative	8.71	Negative
	P62.11	4/13/2018	4 y 1 m	F	HMPV (20)	HMPV	Negative	28.75	Negative
RSV-negative cases	P63.9	4/17/2018	3 y 10 m	M	PIV-1 (38), HMPV (23)	HMPV	Negative	29.19	Negative
	P64.8	4/25/2018	0 y 1 m	M	HMPV (24)	HMPV	Negative	40.37	Negative
	P64.11	4/27/2018	0 y 5 m	M	PIV-3 (35), HMPV (33)	Negative	Negative	27.92	Negative
	P65.12	5/3/2018	3 y 3 m	F	HMPV (30)	HMPV	Negative	48.88	Negative
	P65.18	5/4/2018	0 y 5 m	F	HRV (35), HMPV (23)	HMPV	Negative	15.15	Negative
	P66.4	5/9/2018	2 y 7 m	F	HMPV (25)	HMPV	Negative	17.17	Negative
	P66.13	5/14/2018	0 y 1 m	F	HMPV (26)	HMPV	Negative	33.35	Negative
	P67.2	5/14/2018	2 y 10 m	M	HRV (23), HMPV (22), HBoV 1/2/3/4 (20)	HMPV	Negative	26.06	Negative
	P67.3	5/15/2018	3 y 2 m	M	PIV-3 (32), HRV (31), HMPV (34)	Negative	Negative	44.76	Negative
	P67.7	5/16/2018	1 y 2 m	M	HMPV (29)	HMPV	Negative	24.14	Negative
	P68.3	5/23/2018	1 y 4 m	F	HMPV (31)	HMPV	Negative	26.4	Negative

FICT, Fluorescent immunochromatographic strip test; AD, adenovirus; HRV, Rhinovirus; COV, coronavirus; RSV, respiratory syncytial virus; HEV, Enterovirus; PIV, parainfluenza virus; HBoV, bocavirus; Flu, influenza virus; HMPV, human metapneumovirus; TL, test line; CL, control line. ^a^ SD RSV BIOLINE. ^b^ Cut-off value of FICT (TL/CL) = 53.15.

**Table 2:** Comparison of the clinical diagnostic performance of FICT assay with PCR and commercial RDT.

		RDT			FICT		
		Positive	Negative	Row total	Positive	Negative	Row total
PCR	Positive	15	0	15	18	1	19
	Negative	5	110	115	2	109	111
Column total		20	110	130	20	110	130
% Agreement (kappa)		0.96 [(15 + 110)/130]			0.98 [(18 + 109)/130]		

## Supplementary Materials

The following are available online at http://www.mdpi.com/1422-0067/19/10/3013/s1. Figure S1: Effect of amount of SDS and different pH on E8A11 activity. Different condition of lysis buffer (**A**) and IFA result (**B**). Cells were infected with RSV for 24 h and fixed with 4% paraformaldehyde. After fixation, cells were treated with lysis buffer (1–4) and washed with PBST three times. Fluorescence was detected with a FITC-conjugated secondary antibody. P.C., commercial anti-RSV Fusion antibody. Figure S2: Raw data of FICT assay with antigen. BSA (**A**) and RSV rNP (**B**). Red box indicates each value of TL/CL. Figure S3: Raw data of FICT assay with virus. (**A**) H1N1 and RSV (**B**). Red box indicates each value of TL/CL. Figure S4: Raw data of FICT assay with clinical patients. RSV-negative patients (**A**) and RSV-positive patients (**B**).

## References

[1] Nair H., Nokes D.J., Gessner B.D., Dherani M., Madhi S.A., Singleton R.J., O’Brien K.L., Roca A., Wright P.F., Bruce N. (2010). Global burden of acute lower respiratory infections due to respiratory syncytial virus in young children: A systematic review and meta-analysis. Lancet.

[2] Piedimonte G., Perez M.K. (2014). Respiratory syncytial virus infection and bronchiolitis. Pediatr. Rev..

[3] Macartney K.K., Gorelick M.H., Manning M.L., Hodinka R.L., Bell L.M. (2000). Nosocomial respiratory syncytial virus infections: The cost-effectiveness and cost-benefit of infection control. Pediatrics.

[4] Rima B., Collins P., Easton A., Fouchier R., Kurath G., Lamb R.A., Lee B., Maisner A., Rota P., Wang L. (2017). ICTV Virus Taxonomy Profile: Pneumoviridae. J. Gen. Virol..

[5] Collins P.L., Fearns R., Graham B.S. (2013). Respiratory syncytial virus: Virology, reverse genetics, and pathogenesis of disease. Curr. Topi. Microbiol. Immunol..

[6] Chen Z., Zhang L., Tang A., Callahan C., Pristatsky P., Swoyer R., Cejas P., Nahas D., Galli J., Cosmi S. (2016). Discovery and Characterization of Phage Display-Derived Human Monoclonal Antibodies against RSV F Glycoprotein. PLoS ONE.

[7] Clayton A.L., Albert Z.I., Chantler S.M. (1987). The selection and performance of monoclonal and polyclonal anti-respiratory syncytial virus (RS) antibodies in capture ELISAs for antigen detection. J. Virol. Methods.

[8] Hendry R.M., Godfrey E., Anderson L.J., Fernie B.F., McIntosh K. (1985). Quantification of respiratory syncytial virus polypeptides in nasal secretions by monoclonal antibodies. J. Gen. Virol..

[9] Kanta Subbarao E., Beeler J.A., Waner J.L. (1994). A conformational epitope on the dimer of the fusion protein of respiratory syncytial virus detected in natural infections. Clin. Diagn. Virol..

[10] Routledge E.G., McQuillin J., Samson A.C., Toms G.L. (1985). The development of monoclonal antibodies to respiratory syncytial virus and their use in diagnosis by indirect immunofluorescence. J. Med. Virol..

[11] Wu B. (1985). The production of monoclonal antibodies against respiratory syncytial virus and its clinical applications. Clin. Lab. Med..

[12] Chartrand C., Tremblay N., Renaud C., Papenburg J. (2015). Diagnostic Accuracy of Rapid Antigen Detection Tests for Respiratory Syncytial Virus Infection: Systematic Review and Meta-analysis. J. Clin. Microbiol..

[13] Simabuco F.M., Carromeu C., Farinha-Arcieri L.E., Tamura R.E., Ventura A.M. (2007). Production of polyclonal antibodies against the human respiratory syncytial virus nucleoprotein and phosphoprotein expressed in Escherichia coli. Protein Expr. Purif..

[14] Armbruster D.A., Pry T. (2008). Limit of blank, limit of detection and limit of quantitation. Clin. Biochem. Rev..

[15] McHugh M.L. (2012). Interrater reliability: The kappa statistic. Biochem. Med..

[16] Xu L., Gao H., Zeng J., Liu J., Lu C., Guan X., Qian S., Xie Z. (2018). A fatal case associated with respiratory syncytial virus infection in a young child. BMC Infect. Dis..

[17] Nokes D.J., Okiro E.A., Ngama M., Ochola R., White L.J., Scott P.D., English M., Cane P.A., Medley G.F. (2008). Respiratory syncytial virus infection and disease in infants and young children observed from birth in Kilifi District, Kenya. Clin. Infect. Dis..

[18] Malhotra B., Swamy M.A., Janardhan Reddy P.V., Gupta M.L. (2016). Viruses causing severe acute respiratory infections (SARI) in children ≤5 years of age at a tertiary care hospital in Rajasthan, India. Indian J. Med. Res..

[19] Gómez S., Prieto C., Vera C.R., Otero J., Folgueira L. (2016). Evaluation of a new rapid diagnostic test for the detection of influenza and RSV. Enferm. Infecc. Microbiol. Clin..

[20] Bell D.M., Walsh E.E., Hruska J.F., Schnabel K.C., Hall C.B. (1983). Rapid detection of respiratory syncytial virus with a monoclonal antibody. J. Clin. Microbiol..

[21] Schnee S.V., Pfeil J., Ihling C.M., Tabatabai J., Schnitzler P. (2017). Performance of the Alere i RSV assay for point-of-care detection of respiratory syncytial virus in children. BMC Infect. Dis..

[22] Zhang P., Vemula S.V., Zhao J., Du B., Mohan H., Liu J., El Mubarak H.S., Landry M.L., Hewlett I. (2014). A highly sensitive europium nanoparticle-based immunoassay for detection of influenza A/B virus antigen in clinical specimens. J. Clin. Microbiol..

[23] Schuster J.E., Cox R.G., Hastings A.K., Boyd K.L., Wadia J., Chen Z., Burton D.R., Williamson R.A., Williams J.V. (2015). A broadly neutralizing human monoclonal antibody exhibits in vivo efficacy against both human metapneumovirus and respiratory syncytial virus. J. Infect. Dis..

[24] Corti D., Bianchi S., Vanzetta F., Minola A., Perez L., Agatic G., Guarino B., Silacci C., Marcandalli J., Marsland B.J. (2013). Cross-neutralization of four paramyxoviruses by a human monoclonal antibody. Nature.

[25] Kumari S., Crim R.L., Kulkarni A., Audet S.A., Mdluli T., Murata H., Beeler J.A. (2014). Development of a luciferase immunoprecipitation system assay to detect IgG antibodies against human respiratory syncytial virus nucleoprotein. Clin. Vaccine Immunol..

[26] Percze K., Szakacs Z., Scholz E., Andras J., Szeitner Z., Kieboom C.H., Ferwerda G., Jonge M.I., Gyurcsanyi R.E., Meszaros T. (2017). Aptamers for respiratory syncytial virus detection. Sci. Rep..

[27] Pourianfar H.R., Javadi A., Grollo L. (2012). A colorimetric-based accurate method for the determination of enterovirus 71 titer. Indian J. Virol..

[28] Jung B.K., Choi S.H., Lee J.H., Lee J., Lim C.S. (2016). Performance evaluation of four rapid antigen tests for the detection of Respiratory syncytial virus. J. Med. Virol.

[29] Borg I., Rohde G., Loseke S., Bittscheidt J., Schultze-Werninghaus G., Stephan V., Bufe A. (2003). Evaluation of a quantitative real-time PCR for the detection of respiratory syncytial virus in pulmonary diseases. Eur. Respir. J..

[30] Visseaux B., Collin G., Ichou H., Charpentier C., Bendhafer S., Dumitrescu M., Allal L., Cojocaru B., Desfrère L., Descamps D. (2017). Usefulness of multiplex PCR methods and respiratory viruses’ distribution in children below 15 years old according to age, seasons and clinical units in France: A 3 years retrospective study. PLoS ONE.

[31] Panning M., Hengel H., Henneke P. (2014). The role of multiplex PCR in respiratory tract infections in children. Dtsch. Arztebl. Int..

[32] Mesquita F.D.S., Oliveira D.B.L., Crema D., Pinez C.M.N., Colmanetti T.C., Thomazelli L.M., Gilio A.E., Vieira S.E., Martinez M.B., Botosso V.F. (2017). Rapid antigen detection test for respiratory syncytial virus diagnosis as a diagnostic tool. J. Pediatr..

[33] Wang X.B., He J.S., Fu Y.H., Zheng X.X., Fang X. (2010). Research on the methods for titrating respiratory syncytial virus. Zhonghua Shi Yan He Lin Chuang Bing Du Xue Za Zhi.

[34] Yeo S.J., Bao D.T., Seo G.E., Bui C.T., Kim D.T.H., Anh N.T.V., Tien T.T.T., Linh N.T.P., Sohn H.J., Chong C.K. (2017). Improvement of a rapid diagnostic application of monoclonal antibodies against avian influenza H7 subtype virus using Europium nanoparticles. Sci. Rep..

[35] Yeo S.-J., Liu D.-X., Park H. (2015). Potential Interaction of Plasmodium falciparum Hsp60 and Calpain. Korean J. Parasitol..

[36] Ham J.Y., Jung J., Hwang B.-G., Kim W.-J., Kim Y.-S., Kim E.-J., Cho M.-Y., Hwang M.-S., Won D.I., Suh J.S. (2015). Highly sensitive and novel point-of-care system, aQcare Chlamydia TRF kit for detecting Chlamydia trachomatis by using europium (Eu)(III) chelated nanoparticles. Ann. Lab. Med..

[37] Do L.A., van Doorn H.R., Bryant J.E., Nghiem M.N., Nguyen Van V.C., Vo C.K., Nguyen M.D., Tran T.H., Farrar J., de Jong M.D. (2012). A sensitive real-time PCR for detection and subgrouping of human respiratory syncytial virus. J. Virol. Methods.

