# Supplementary material for: Development of a Rapid Fluorescent Immunochromatographic Test to Detect Respiratory Syncytial Virus
Source: Int J Mol Sci. 2018 Oct 2;19(10):3013. doi: 10.3390/ijms19103013 (PMC6212954; doi:10.3390/ijms19103013)
Supplement: Supplementary file 1 [file ijms-19-03013-s001.pdf]

Supplementary materials

A

Lysis buffer	Tris	EDTA	Triton X-100	SDS	pH
1	0.1 M	0.1 M	1%	0.2%	5
2	0.1 M	0.1 M	1%	0.2%	8
3	0.1 M	0.1 M	1%	1%	8
4	0.1 M	0.1 M	1%	1%	9

B

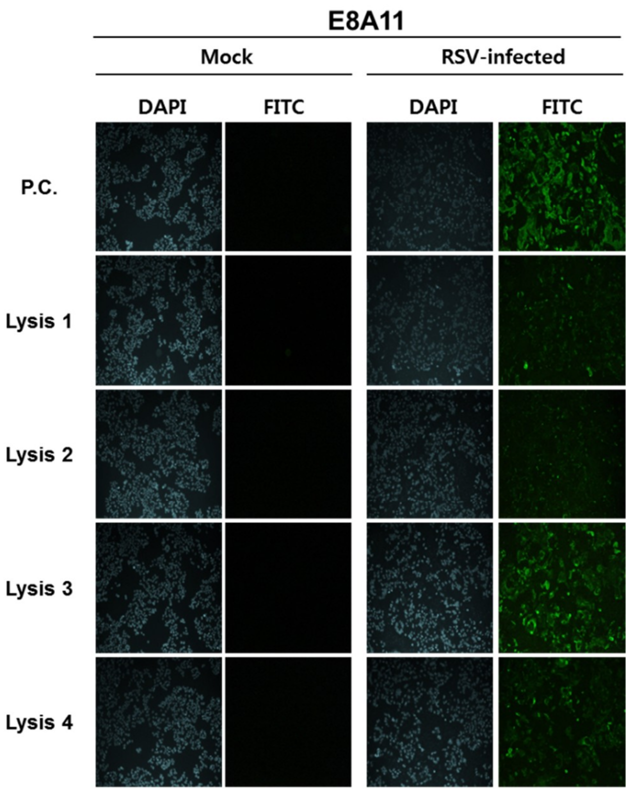

Figure S1. Effect of amount of SDS and different pH on E8A11 activity. Different condition of lysis buffer (A) and IFA result (B). Cells were infected with RSV for 24 h and fixed with 4% paraformaldehyde. After fixation, cells were treated with lysis buffer (1-4) and washed with PBST three times. Fluorescence was detected with a FITC-conjugated secondary antibody. P.C., commercial anti-RSV Fusion antibody

A

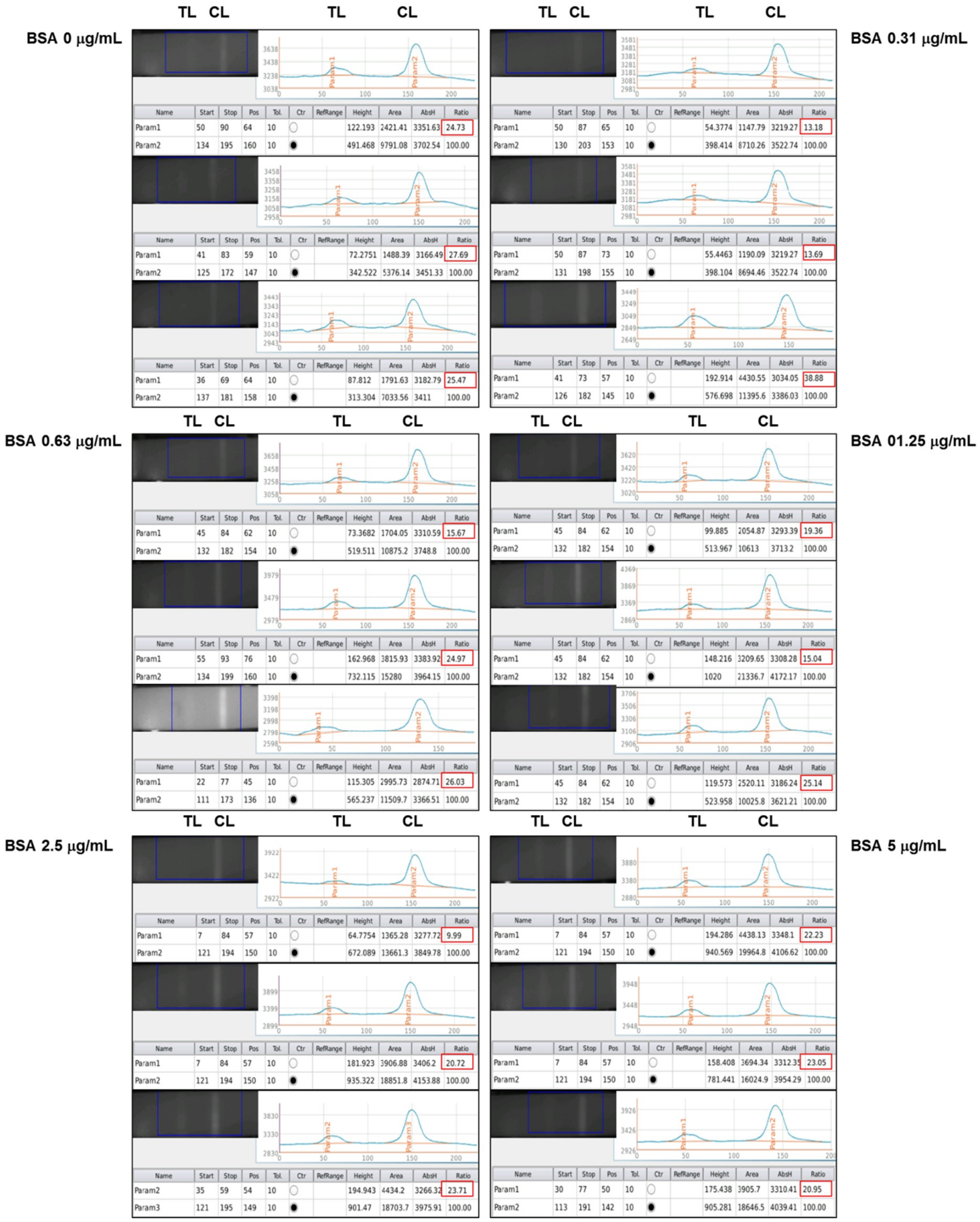

BSA 0 μg/mL

Name	Start	Stop	Pos	Tol.	Ctr	RefRange	Height	Area	AbsH	Ratio
Param1	50	90	64	10	○		122.193	2421.41	3351.63	24.73
Param2	134	195	160	10	●		491.468	9791.08	3702.54	100.00

Name	Start	Stop	Pos	Tol.	Ctr	RefRange	Height	Area	AbsH	Ratio
Param1	41	83	59	10	○		72.2751	1488.39	3166.49	27.69
Param2	125	172	147	10	●		342.522	5376.14	3451.33	100.00

Name	Start	Stop	Pos	Tol.	Ctr	RefRange	Height	Area	AbsH	Ratio
Param1	36	69	64	10	○		87.812	1791.63	3182.79	25.47
Param2	137	181	158	10	●		313.304	7033.56	3411	100.00

BSA 0.31 μg/mL

Name	Start	Stop	Pos	Tol.	Ctr	RefRange	Height	Area	AbsH	Ratio
Param1	50	87	65	10	○		54.3774	1147.79	3219.27	13.18
Param2	130	203	153	10	●		398.414	8710.26	3522.74	100.00

Name	Start	Stop	Pos	Tol.	Ctr	RefRange	Height	Area	AbsH	Ratio
Param1	50	87	73	10	○		55.4463	1190.09	3219.27	13.69
Param2	131	198	155	10	●		398.104	8694.46	3522.74	100.00

Name	Start	Stop	Pos	Tol.	Ctr	RefRange	Height	Area	AbsH	Ratio
Param1	41	73	57	10	○		192.914	4430.55	3034.05	38.88
Param2	126	182	145	10	●		576.698	11395.6	3386.03	100.00

BSA 0.63 μg/mL

Name	Start	Stop	Pos	Tol.	Ctr	RefRange	Height	Area	AbsH	Ratio
Param1	45	84	62	10	○		73.3682	1704.05	3310.59	15.67
Param2	132	182	154	10	●		519.511	10875.2	3748.8	100.00

Name	Start	Stop	Pos	Tol.	Ctr	RefRange	Height	Area	AbsH	Ratio
Param1	55	93	76	10	○		162.968	3815.93	3383.92	24.97
Param2	134	199	160	10	●		732.115	15280	3964.15	100.00

Name	Start	Stop	Pos	Tol.	Ctr	RefRange	Height	Area	AbsH	Ratio
Param1	22	77	45	10	○		115.305	2995.73	2874.71	26.03
Param2	111	173	136	10	●		565.237	11509.7	3366.51	100.00

BSA 01.25 μg/mL

Name	Start	Stop	Pos	Tol.	Ctr	RefRange	Height	Area	AbsH	Ratio
Param1	45	84	62	10	○		99.885	2054.87	3293.39	19.36
Param2	132	182	154	10	●		513.967	10613	3713.2	100.00

Name	Start	Stop	Pos	Tol.	Ctr	RefRange	Height	Area	AbsH	Ratio
Param1	45	84	62	10	○		148.216	3209.65	3308.28	15.04
Param2	132	182	154	10	●		1020	21336.7	4172.17	100.00

Name	Start	Stop	Pos	Tol.	Ctr	RefRange	Height	Area	AbsH	Ratio
Param1	45	84	62	10	○		119.573	2520.11	3186.24	25.14
Param2	132	182	154	10	●		523.958	10025.8	3621.21	100.00

BSA 2.5 μg/mL

Name	Start	Stop	Pos	Tol.	Ctr	RefRange	Height	Area	AbsH	Ratio
Param1	7	84	57	10	○		64.7754	1365.28	3277.72	9.99
Param2	121	194	150	10	●		672.089	13661.3	3849.78	100.00

Name	Start	Stop	Pos	Tol.	Ctr	RefRange	Height	Area	AbsH	Ratio
Param1	7	84	57	10	○		181.923	3906.88	3406.2	20.72
Param2	121	194	150	10	●		935.322	18851.8	4153.88	100.00

Name	Start	Stop	Pos	Tol.	Ctr	RefRange	Height	Area	AbsH	Ratio
Param2	35	59	54	10	○		194.943	4434.2	3266.32	23.71
Param3	121	195	149	10	●		901.47	18703.7	3975.91	100.00

BSA 5 μg/mL

Name	Start	Stop	Pos	Tol.	Ctr	RefRange	Height	Area	AbsH	Ratio
Param1	7	84	57	10	○		194.286	4438.13	3348.1	22.23
Param2	121	194	150	10	●		940.569	19964.8	4106.62	100.00

Name	Start	Stop	Pos	Tol.	Ctr	RefRange	Height	Area	AbsH	Ratio
Param1	7	84	57	10	○		158.408	3694.34	3312.35	23.05
Param2	121	194	150	10	●		781.441	16024.9	3954.29	100.00

Name	Start	Stop	Pos	Tol.	Ctr	RefRange	Height	Area	AbsH	Ratio
Param1	30	77	50	10	○		175.438	3905.7	3310.41	20.95
Param2	113	191	142	10	●		905.281	18646.5	4039.41	100.00

B

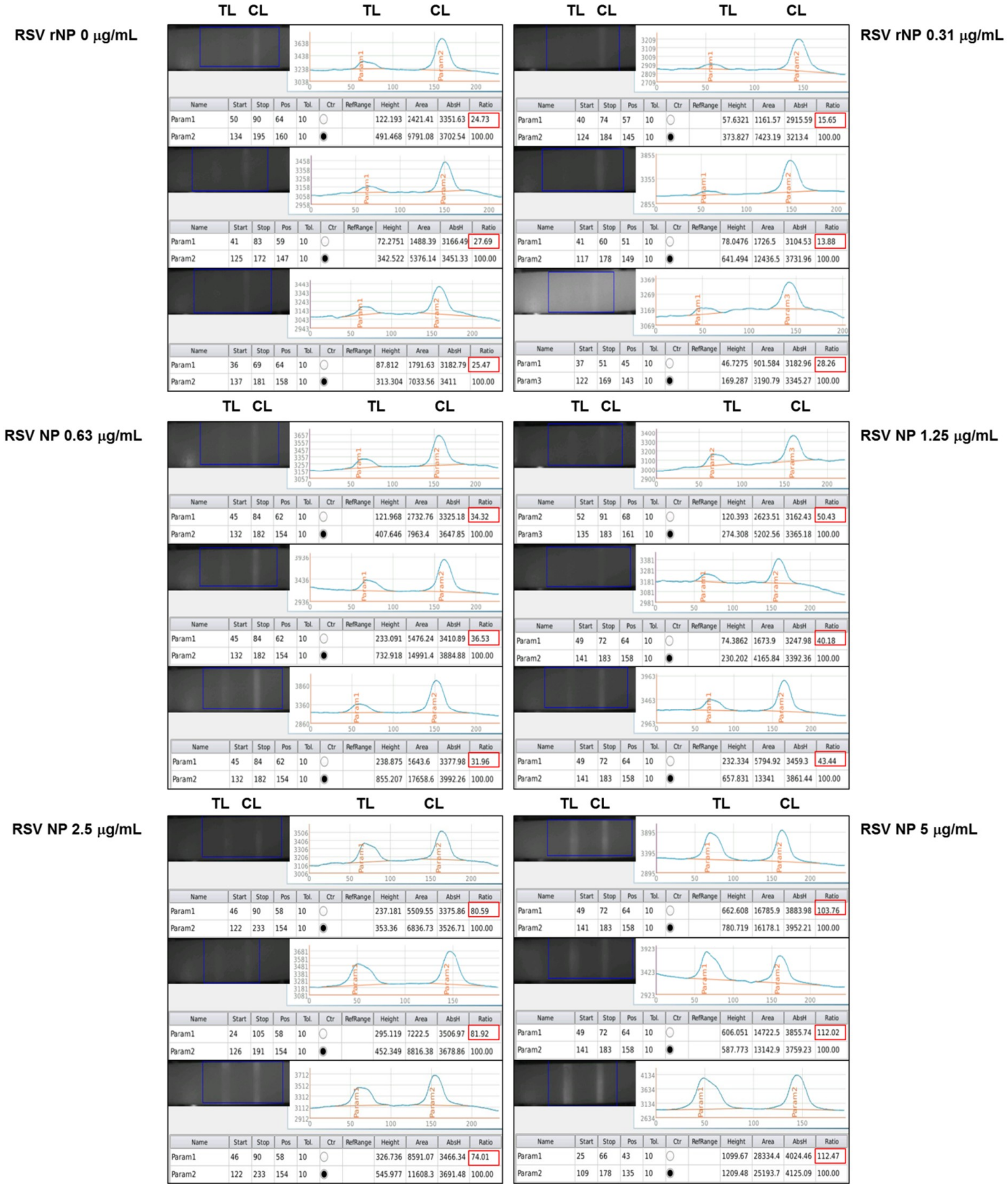

Figure S2. Raw data of FICT assay with antigen. BSA (A) and RSV rNP(B). Red box indicates each value of TL/CL.

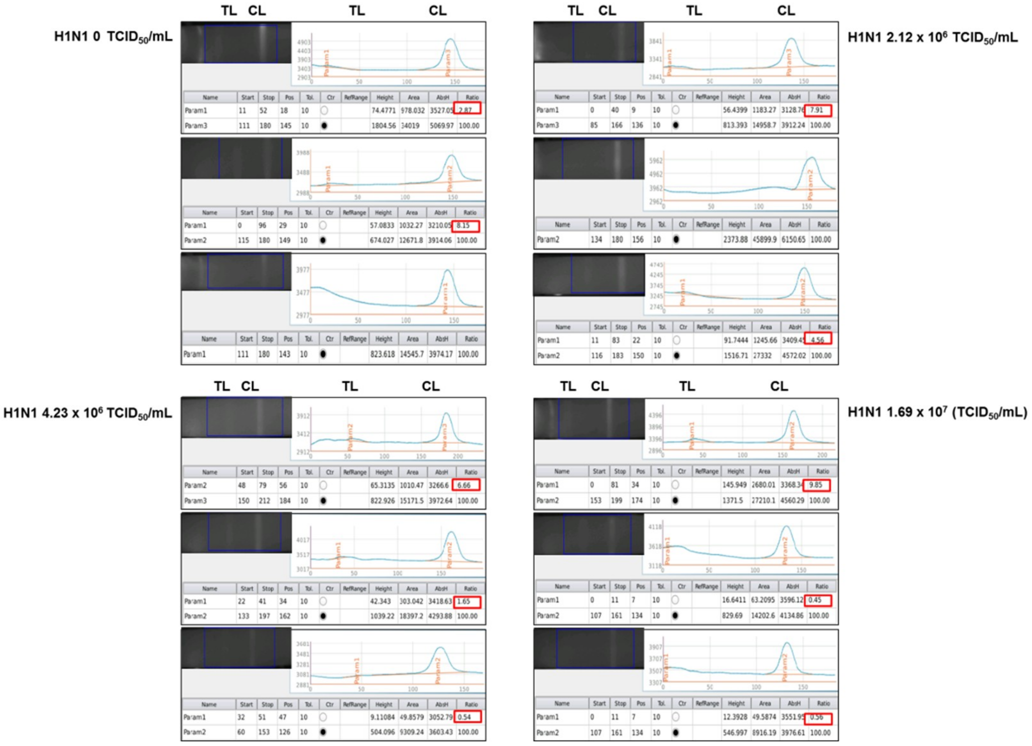

B

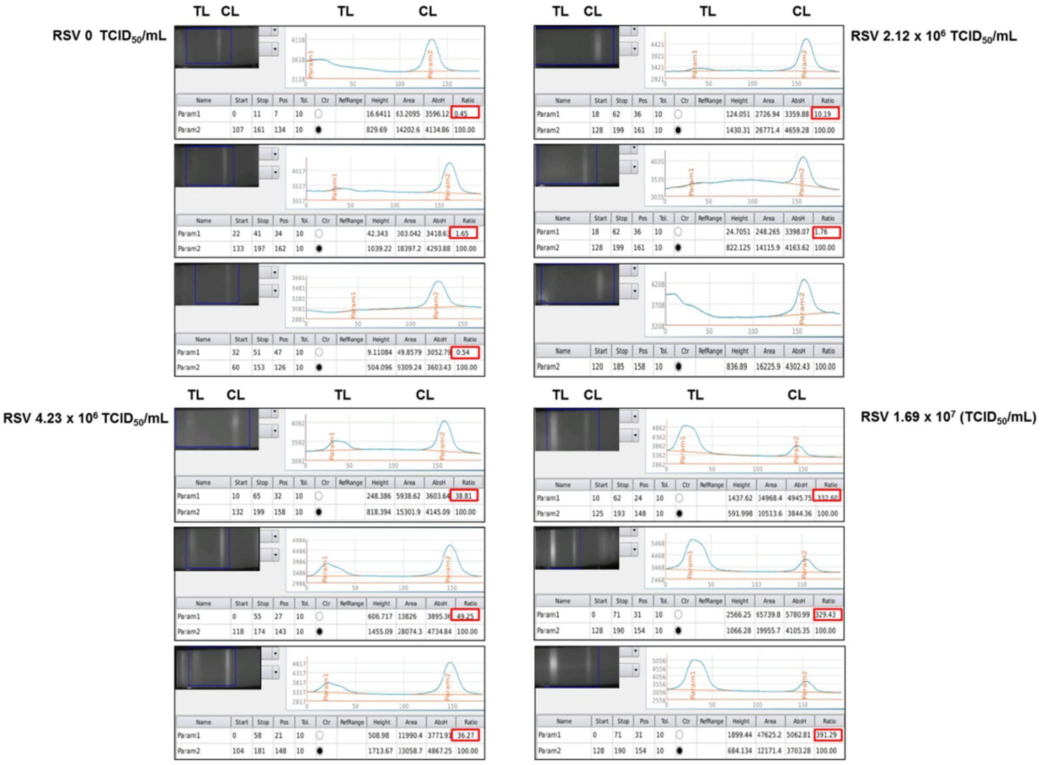

Figure S3. Raw data of FICT assay with virus. (A) H1N1 and RSV(B). Red box indicates each value of TL/CL.

A

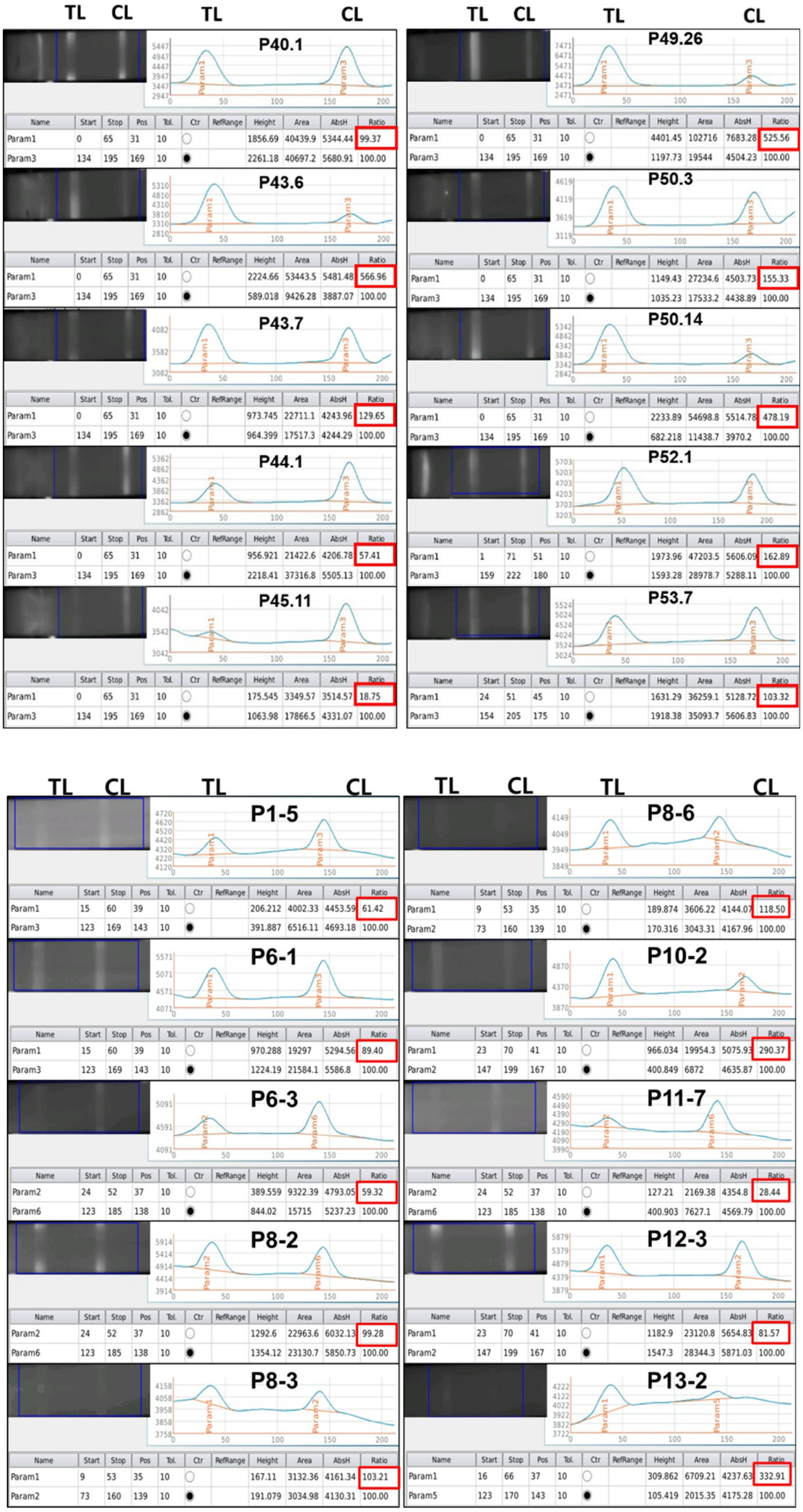
TL
CL
P40.1
P49.26
P43.6
P50.3
P43.7
P50.14
P44.1
P52.1
P45.11
P53.7
P1-5
P8-6
P6-1
P10-2
P6-3
P11-7
P8-2
P12-3
P8-3
P13-2

B

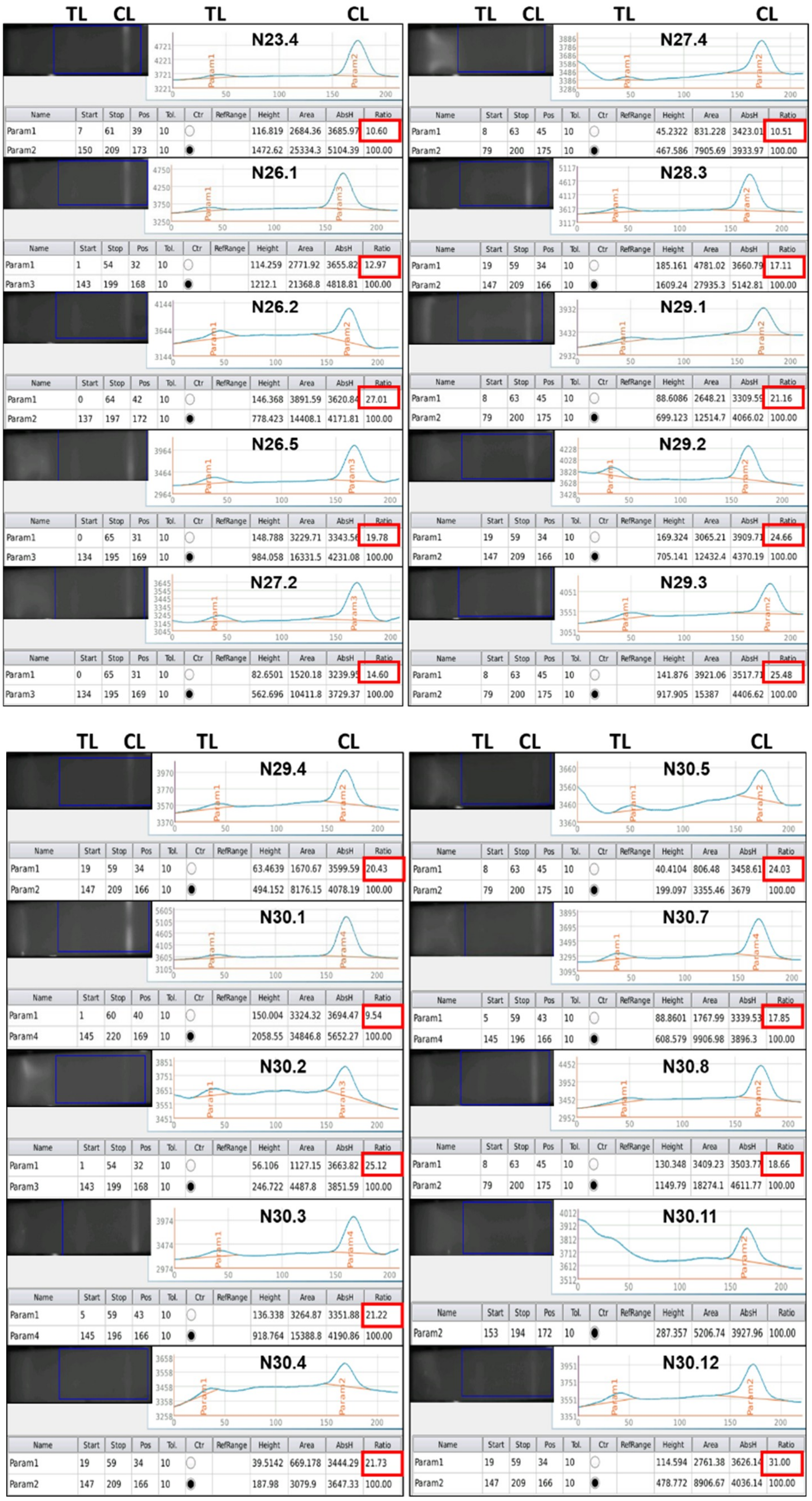
TL CL TL CL
N23.4
Name Start Stop Pos Tol. Ctr RefRange Height Area AbsH Ratio
Param1 7 61 39 10 116.819 2684.36 3685.97 10.60
Param2 150 209 173 10 1472.62 25334.3 5104.39 100.00
N26.1
Param1 1 54 32 10 114.259 2771.92 3655.82 12.97
Param3 143 199 168 10 1212.1 21368.8 4818.81 100.00
N26.2
Param1 0 64 42 10 146.368 3891.59 3620.84 27.01
Param2 137 197 172 10 778.423 14408.1 4171.81 100.00
N26.5
Param1 0 65 31 10 148.788 3229.71 3343.56 19.78
Param3 134 195 169 10 984.058 16331.5 4231.08 100.00
N27.2
Param1 0 65 31 10 82.6501 1520.18 3239.95 14.60
Param3 134 195 169 10 562.696 10411.8 3729.37 100.00
N27.4
Param1 8 63 45 10 45.2322 831.228 3423.01 10.51
Param2 79 200 175 10 467.586 7905.69 3933.97 100.00
N28.3
Param1 19 59 34 10 185.161 4781.02 3660.79 17.11
Param2 147 209 166 10 1609.24 27935.3 5142.81 100.00
N29.1
Param1 8 63 45 10 88.6086 2648.21 3309.59 21.16
Param2 79 200 175 10 699.123 12514.7 4066.02 100.00
N29.2
Param1 19 59 34 10 169.324 3065.21 3909.71 24.66
Param2 147 209 166 10 705.141 12432.4 4370.19 100.00
N29.3
Param1 8 63 45 10 141.876 3921.06 3517.71 25.48
Param2 79 200 175 10 917.905 15387 4406.62 100.00
N29.4
Param1 19 59 34 10 63.4639 1670.67 3599.59 20.43
Param2 147 209 166 10 494.152 8176.15 4078.19 100.00
N30.1
Param1 1 60 40 10 150.004 3324.32 3694.47 9.54
Param4 145 220 169 10 2058.55 34846.8 5652.27 100.00
N30.2
Param1 1 54 32 10 56.106 1127.15 3663.82 25.12
Param3 143 199 168 10 246.722 4487.8 3851.59 100.00
N30.3
Param1 5 59 43 10 136.338 3264.87 3351.88 21.22
Param4 145 196 166 10 918.764 15388.8 4190.86 100.00
N30.4
Param1 19 59 34 10 39.5142 669.178 3444.29 21.73
Param2 147 209 166 10 187.98 3079.9 3647.33 100.00
N30.5
Param1 8 63 45 10 40.4104 806.48 3458.61 24.03
Param2 79 200 175 10 199.097 3355.46 3679 100.00
N30.7
Param1 5 59 43 10 88.8601 1767.99 3339.53 17.85
Param4 145 196 166 10 608.579 9906.98 3896.3 100.00
N30.8
Param1 8 63 45 10 130.348 3409.23 3503.77 18.66
Param2 79 200 175 10 1149.79 18274.1 4611.77 100.00
N30.11
Param2 153 194 172 10 287.357 5206.74 3927.96 100.00
N30.12
Param1 19 59 34 10 114.594 2761.38 3626.14 31.00
Param2 147 209 166 10 478.772 8906.67 4036.14 100.00

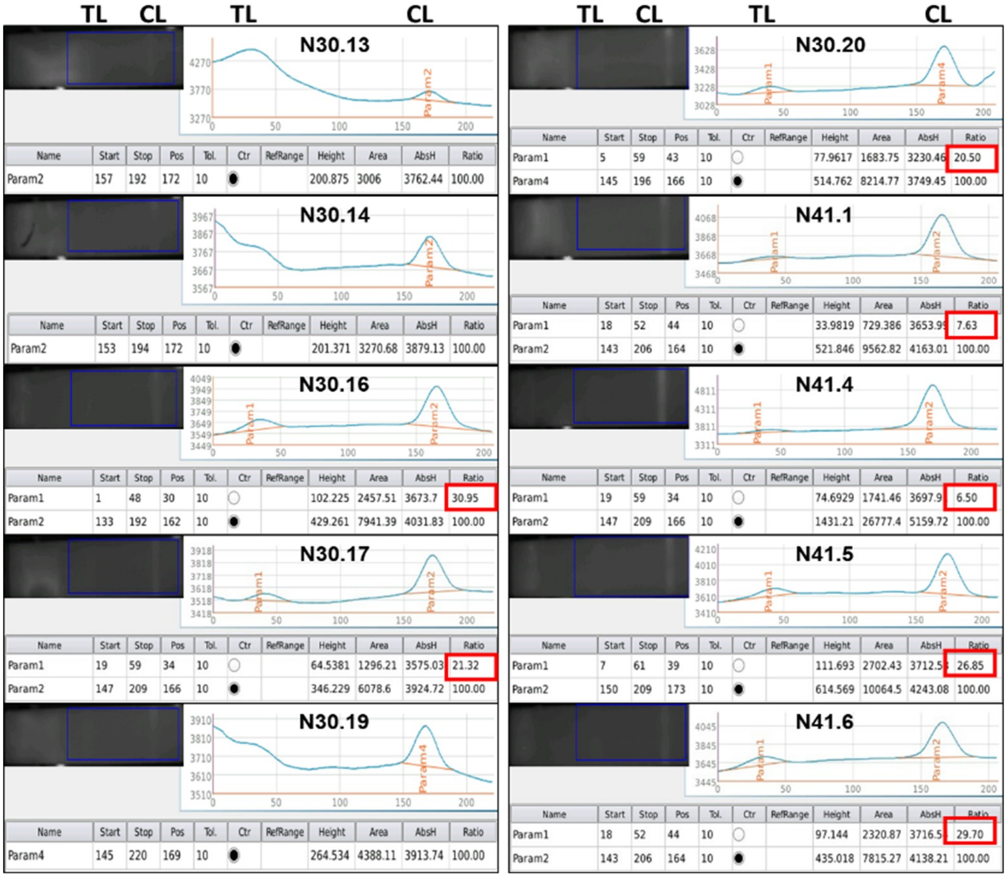
TL CL TL CL
N30.13
Name Start Stop Pos Tol. Ctr RefRange Height Area AbsH Ratio
Param2 157 192 172 10 200.875 3006 3762.44 100.00
N30.14
Param2 153 194 172 10 201.371 3270.68 3879.13 100.00
N30.16
Param1 1 48 30 10 102.225 2457.51 3673.7 30.95
Param2 133 192 162 10 429.261 7941.39 4031.83 100.00
N30.17
Param1 19 59 34 10 64.5381 1296.21 3575.03 21.32
Param2 147 209 166 10 346.229 6078.6 3924.72 100.00
N30.19
Param4 145 220 169 10 264.534 4388.11 3913.74 100.00
N30.20
Param1 5 59 43 10 77.9617 1683.75 3230.46 20.50
Param4 145 196 166 10 514.762 8214.77 3749.45 100.00
N41.1
Param1 18 52 44 10 33.9819 729.386 3653.9 7.63
Param2 143 206 164 10 521.846 9562.82 4163.01 100.00
N41.4
Param1 19 59 34 10 74.6929 1741.46 3697.9 6.50
Param2 147 209 166 10 1431.21 26777.4 5159.72 100.00
N41.5
Param1 7 61 39 10 111.693 2702.43 3712.5 26.85
Param2 150 209 173 10 614.569 10064.5 4243.08 100.00
N41.6
Param1 18 52 44 10 97.144 2320.87 3716.5 29.70
Param2 143 206 164 10 435.018 7815.27 4138.21 100.00

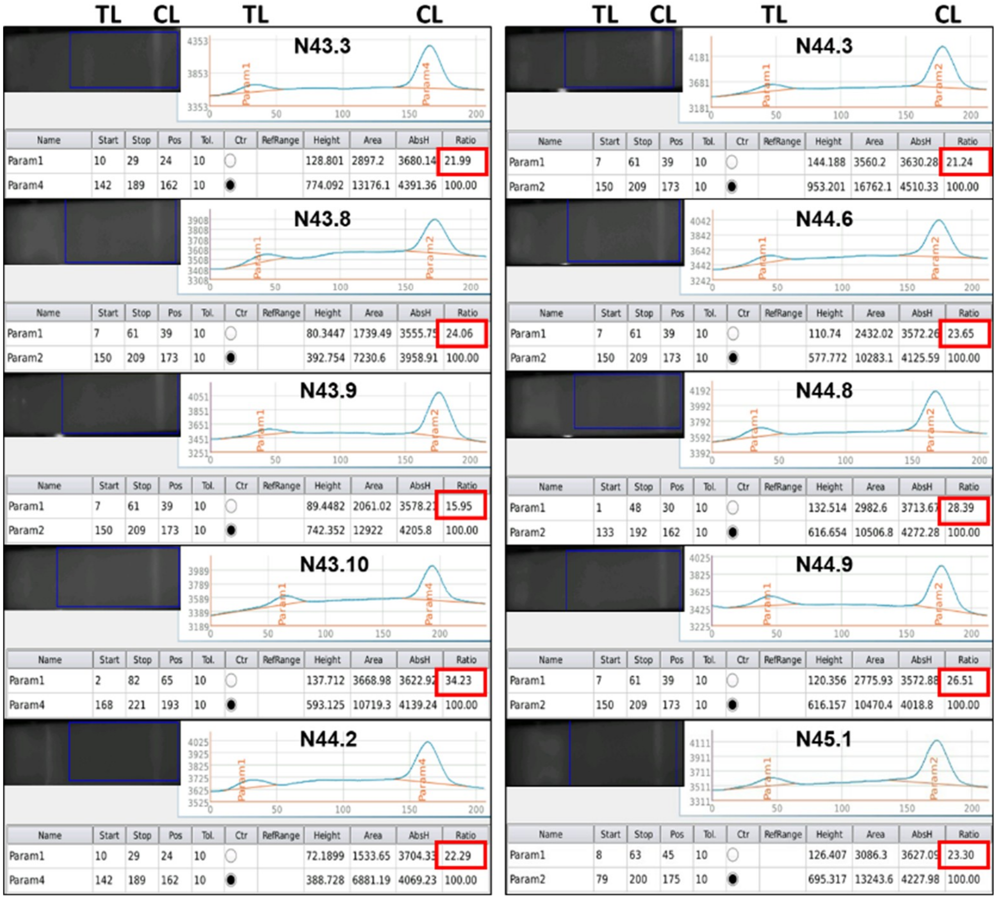
TL CL TL CL
N43.3
Name Start Stop Pos Tol. Ctr RefRange Height Area AbsH Ratio
Param1 10 29 24 10 128.801 2897.2 3680.14 21.99
Param4 142 189 162 10 774.092 13176.1 4391.36 100.00
N43.8
Param1 7 61 39 10 80.3447 1739.49 3555.75 24.06
Param2 150 209 173 10 392.754 7230.6 3958.91 100.00
N43.9
Param1 7 61 39 10 89.4482 2061.02 3578.21 15.95
Param2 150 209 173 10 742.352 12922 4205.8 100.00
N43.10
Param1 2 82 65 10 137.712 3668.98 3622.92 34.23
Param4 168 221 193 10 593.125 10719.3 4139.24 100.00
N44.2
Param1 10 29 24 10 72.1899 1533.65 3704.33 22.29
Param4 142 189 162 10 388.728 6881.19 4069.23 100.00
N44.3
Param1 7 61 39 10 144.188 3560.2 3630.28 21.24
Param2 150 209 173 10 953.201 16762.1 4510.33 100.00
N44.6
Param1 7 61 39 10 110.74 2432.02 3572.26 23.65
Param2 150 209 173 10 577.772 10283.1 4125.59 100.00
N44.8
Param1 1 48 30 10 132.514 2982.6 3713.67 28.39
Param2 133 192 162 10 616.654 10506.8 4272.28 100.00
N44.9
Param1 7 61 39 10 120.356 2775.93 3572.88 26.51
Param2 150 209 173 10 616.157 10470.4 4018.8 100.00
N45.1
Param1 8 63 45 10 126.407 3086.3 3627.09 23.30
Param2 79 200 175 10 695.317 13243.6 4227.98 100.00

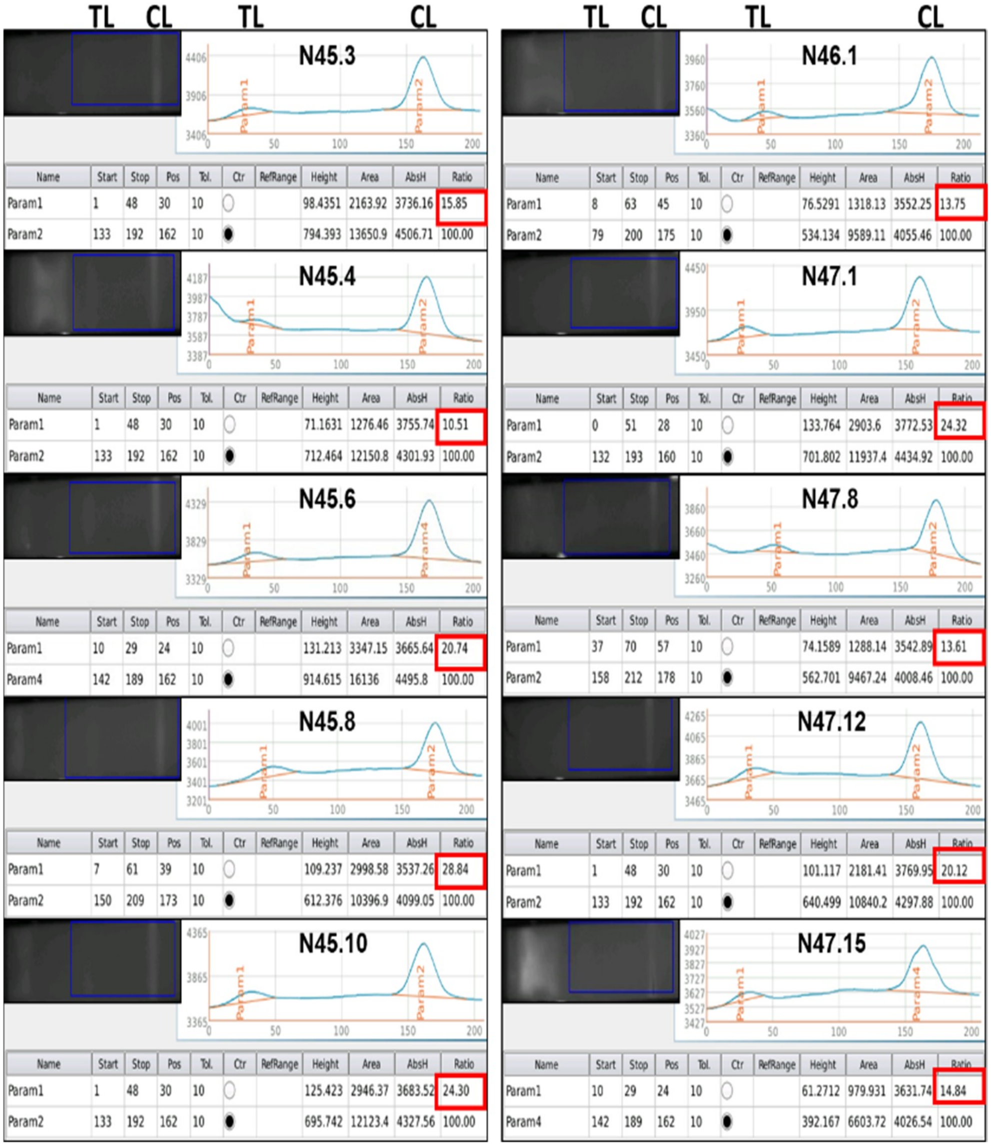

N45.3

Name	Start	Stop	Pos	Tol.	Ctr	RefRange	Height	Area	AbsH	Ratio
Param1	1	48	30	10	○		98.4351	2163.92	3736.16	15.85
Param2	133	192	162	10	●		794.393	13650.9	4506.71	100.00

N46.1

Name	Start	Stop	Pos	Tol.	Ctr	RefRange	Height	Area	AbsH	Ratio
Param1	8	63	45	10	○		76.5291	1318.13	3552.25	13.75
Param2	79	200	175	10	●		534.134	9589.11	4055.46	100.00

N45.4

Name	Start	Stop	Pos	Tol.	Ctr	RefRange	Height	Area	AbsH	Ratio
Param1	1	48	30	10	○		71.1631	1276.46	3755.74	10.51
Param2	133	192	162	10	●		712.464	12150.8	4301.93	100.00

N47.1

Name	Start	Stop	Pos	Tol.	Ctr	RefRange	Height	Area	AbsH	Ratio
Param1	0	51	28	10	○		133.764	2903.6	3772.53	24.32
Param2	132	193	160	10	●		701.802	11937.4	4434.92	100.00

N45.6

Name	Start	Stop	Pos	Tol.	Ctr	RefRange	Height	Area	AbsH	Ratio
Param1	10	29	24	10	○		131.213	3347.15	3665.64	20.74
Param4	142	189	162	10	●		914.615	16136	4495.8	100.00

N47.8

Name	Start	Stop	Pos	Tol.	Ctr	RefRange	Height	Area	AbsH	Ratio
Param1	37	70	57	10	○		74.1589	1288.14	3542.89	13.61
Param2	158	212	178	10	●		562.701	9467.24	4008.46	100.00

N45.8

Name	Start	Stop	Pos	Tol.	Ctr	RefRange	Height	Area	AbsH	Ratio
Param1	7	61	39	10	○		109.237	2998.58	3537.26	28.84
Param2	150	209	173	10	●		612.376	10396.9	4099.05	100.00

N47.12

Name	Start	Stop	Pos	Tol.	Ctr	RefRange	Height	Area	AbsH	Ratio
Param1	1	48	30	10	○		101.117	2181.41	3769.95	20.12
Param2	133	192	162	10	●		640.499	10840.2	4297.88	100.00

N45.10

Name	Start	Stop	Pos	Tol.	Ctr	RefRange	Height	Area	AbsH	Ratio
Param1	1	48	30	10	○		125.423	2946.37	3683.52	24.30
Param2	133	192	162	10	●		695.742	12123.4	4327.56	100.00

N47.15

Name	Start	Stop	Pos	Tol.	Ctr	RefRange	Height	Area	AbsH	Ratio
Param1	10	29	24	10	○		61.2712	979.931	3631.74	14.84
Param4	142	189	162	10	●		392.167	6603.72	4026.54	100.00

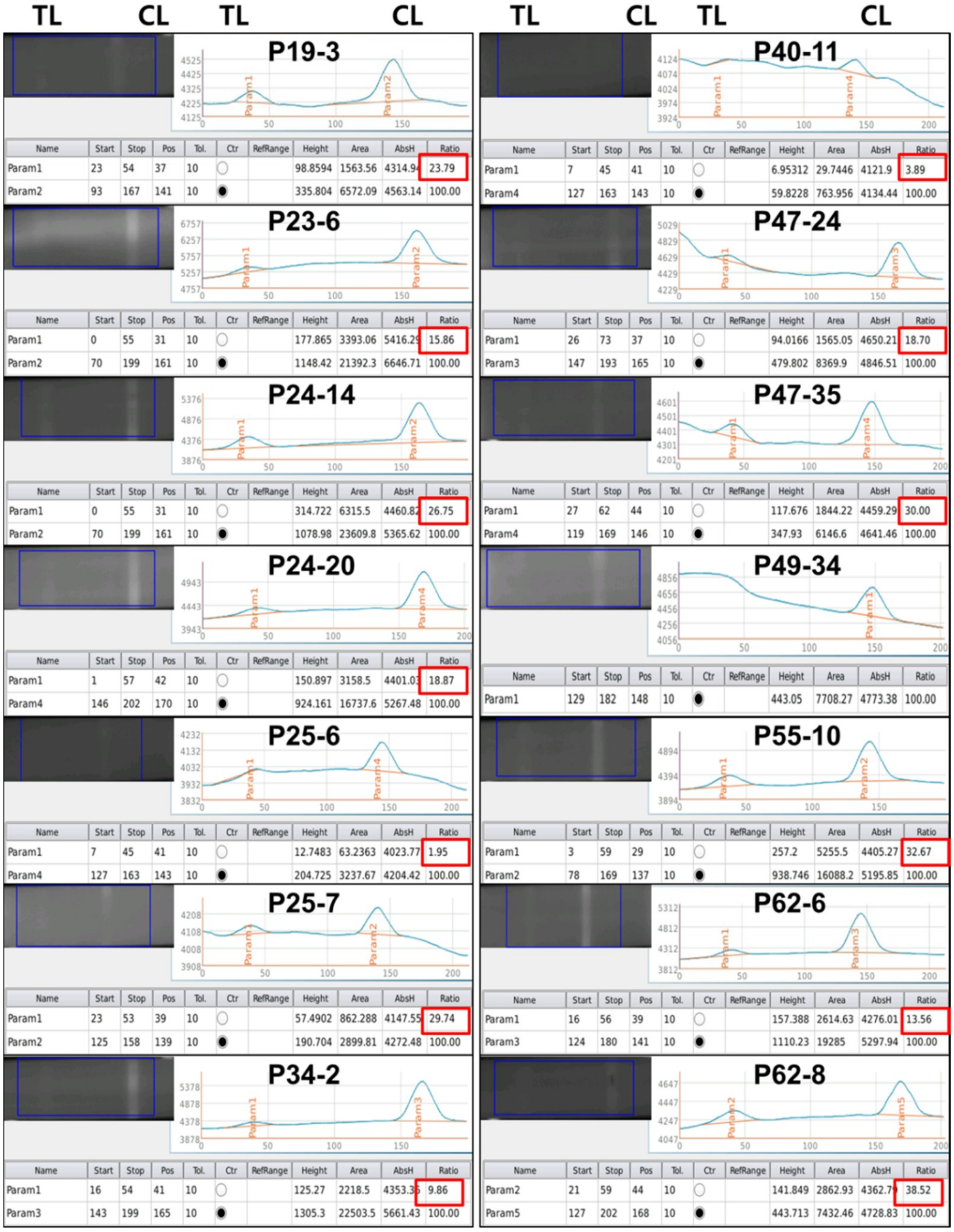
TL CL TL CL
TL CL TL CL
P19-3
Name Start Stop Pos Tol. Ctr RefRange Height Area AbsH Ratio
Param1 23 54 37 10 98.8594 1563.56 4314.94 23.79
Param2 93 167 141 10 335.804 6572.09 4563.14 100.00
P40-11
Name Start Stop Pos Tol. Ctr RefRange Height Area AbsH Ratio
Param1 7 45 41 10 6.95312 29.7446 4121.9 3.89
Param4 127 163 143 10 59.8228 763.956 4134.44 100.00
P23-6
Name Start Stop Pos Tol. Ctr RefRange Height Area AbsH Ratio
Param1 0 55 31 10 177.865 3393.06 5416.29 15.86
Param2 70 199 161 10 1148.42 21392.3 6646.71 100.00
P47-24
Name Start Stop Pos Tol. Ctr RefRange Height Area AbsH Ratio
Param1 26 73 37 10 94.0166 1565.05 4650.21 18.70
Param3 147 193 165 10 479.802 8369.9 4846.51 100.00
P24-14
Name Start Stop Pos Tol. Ctr RefRange Height Area AbsH Ratio
Param1 0 55 31 10 314.722 6315.5 4460.82 26.75
Param2 70 199 161 10 1078.98 23609.8 5365.62 100.00
P47-35
Name Start Stop Pos Tol. Ctr RefRange Height Area AbsH Ratio
Param1 27 62 44 10 117.676 1844.22 4459.29 30.00
Param4 119 169 146 10 347.93 6146.6 4641.46 100.00
P24-20
Name Start Stop Pos Tol. Ctr RefRange Height Area AbsH Ratio
Param1 1 57 42 10 150.897 3158.5 4401.03 18.87
Param4 146 202 170 10 924.161 16737.6 5267.48 100.00
P49-34
Name Start Stop Pos Tol. Ctr RefRange Height Area AbsH Ratio
Param1 129 182 148 10 443.05 7708.27 4773.38 100.00
P25-6
Name Start Stop Pos Tol. Ctr RefRange Height Area AbsH Ratio
Param1 7 45 41 10 12.7483 63.2363 4023.77 1.95
Param4 127 163 143 10 204.725 3237.67 4204.42 100.00
P55-10
Name Start Stop Pos Tol. Ctr RefRange Height Area AbsH Ratio
Param1 3 59 29 10 257.2 5255.5 4405.27 32.67
Param2 78 169 137 10 938.746 16088.2 5195.85 100.00
P25-7
Name Start Stop Pos Tol. Ctr RefRange Height Area AbsH Ratio
Param1 23 53 39 10 57.4902 862.288 4147.55 29.74
Param2 125 158 139 10 190.704 2899.81 4272.48 100.00
P62-6
Name Start Stop Pos Tol. Ctr RefRange Height Area AbsH Ratio
Param1 16 56 39 10 157.388 2614.63 4276.01 13.56
Param3 124 180 141 10 1110.23 19285 5297.94 100.00
P34-2
Name Start Stop Pos Tol. Ctr RefRange Height Area AbsH Ratio
Param1 16 54 41 10 125.27 2218.5 4353.35 9.86
Param3 143 199 165 10 1305.3 22503.5 5661.43 100.00
P62-8
Name Start Stop Pos Tol. Ctr RefRange Height Area AbsH Ratio
Param2 21 59 44 10 141.849 2862.93 4362.79 38.52
Param5 127 202 168 10 443.713 7432.46 4728.83 100.00

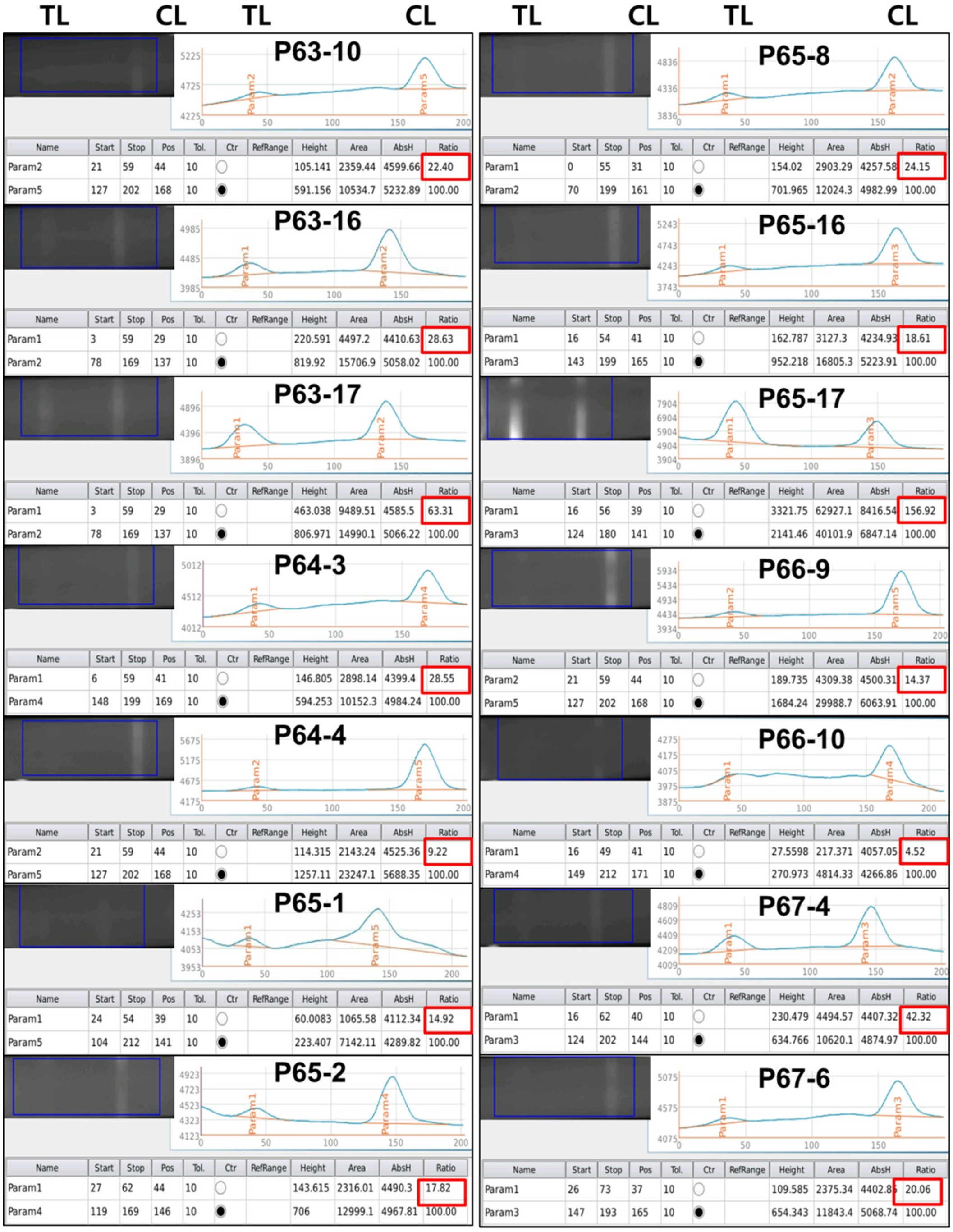

P63-10

Name	Start	Stop	Pos	Tol.	Ctr	RefRange	Height	Area	AbsH	Ratio
Param2	21	59	44	10	○		105.141	2359.44	4599.66	22.40
Param5	127	202	168	10	●		591.156	10534.7	5232.89	100.00

P63-16

Name	Start	Stop	Pos	Tol.	Ctr	RefRange	Height	Area	AbsH	Ratio
Param1	3	59	29	10	○		220.591	4497.2	4410.63	28.63
Param2	78	169	137	10	●		819.92	15706.9	5058.02	100.00

P63-17

Name	Start	Stop	Pos	Tol.	Ctr	RefRange	Height	Area	AbsH	Ratio
Param1	3	59	29	10	○		463.038	9489.51	4585.5	63.31
Param2	78	169	137	10	●		806.971	14990.1	5066.22	100.00

P64-3

Name	Start	Stop	Pos	Tol.	Ctr	RefRange	Height	Area	AbsH	Ratio
Param1	6	59	41	10	○		146.805	2898.14	4399.4	28.55
Param4	148	199	169	10	●		594.253	10152.3	4984.24	100.00

P64-4

Name	Start	Stop	Pos	Tol.	Ctr	RefRange	Height	Area	AbsH	Ratio
Param2	21	59	44	10	○		114.315	2143.24	4525.36	9.22
Param5	127	202	168	10	●		1257.11	23247.1	5688.35	100.00

P65-1

Name	Start	Stop	Pos	Tol.	Ctr	RefRange	Height	Area	AbsH	Ratio
Param1	24	54	39	10	○		60.0083	1065.58	4112.34	14.92
Param5	104	212	141	10	●		223.407	7142.11	4289.82	100.00

P65-2

Name	Start	Stop	Pos	Tol.	Ctr	RefRange	Height	Area	AbsH	Ratio
Param1	27	62	44	10	○		143.615	2316.01	4490.3	17.82
Param4	119	169	146	10	●		706	12999.1	4967.81	100.00

P65-8

Name	Start	Stop	Pos	Tol.	Ctr	RefRange	Height	Area	AbsH	Ratio
Param1	0	55	31	10	○		154.02	2903.29	4257.58	24.15
Param2	70	199	161	10	●		701.965	12024.3	4982.99	100.00

P65-16

Name	Start	Stop	Pos	Tol.	Ctr	RefRange	Height	Area	AbsH	Ratio
Param1	16	54	41	10	○		162.787	3127.3	4234.93	18.61
Param3	143	199	165	10	●		952.218	16805.3	5223.91	100.00

P65-17

Name	Start	Stop	Pos	Tol.	Ctr	RefRange	Height	Area	AbsH	Ratio
Param1	16	56	39	10	○		3321.75	62927.1	8416.54	156.92
Param3	124	180	141	10	●		2141.46	40101.9	6847.14	100.00

P66-9

Name	Start	Stop	Pos	Tol.	Ctr	RefRange	Height	Area	AbsH	Ratio
Param2	21	59	44	10	○		189.735	4309.38	4500.31	14.37
Param5	127	202	168	10	●		1684.24	29988.7	6063.91	100.00

P66-10

Name	Start	Stop	Pos	Tol.	Ctr	RefRange	Height	Area	AbsH	Ratio
Param1	16	49	41	10	○		27.5598	217.371	4057.05	4.52
Param4	149	212	171	10	●		270.973	4814.33	4266.86	100.00

P67-4

Name	Start	Stop	Pos	Tol.	Ctr	RefRange	Height	Area	AbsH	Ratio
Param1	16	62	40	10	○		230.479	4494.57	4407.32	42.32
Param3	124	202	144	10	●		634.766	10620.1	4874.97	100.00

P67-6

Name	Start	Stop	Pos	Tol.	Ctr	RefRange	Height	Area	AbsH	Ratio
Param1	26	73	37	10	○		109.585	2375.34	4402.8[illegible]	20.06
Param3	147	193	165	10	●		654.343	11843.4	5068.74	100.00

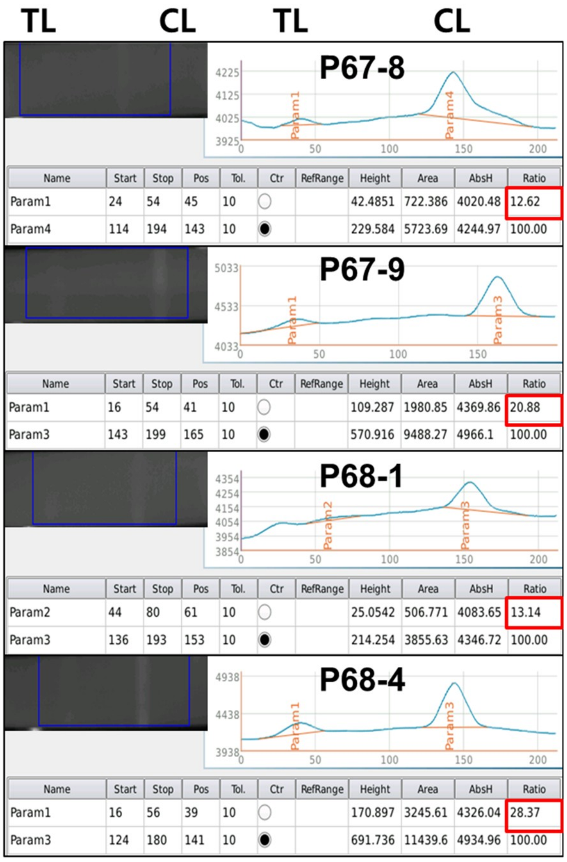

Figure S4. Raw data of FICT assay with clinical patients. RSV-negative patients (A) and RSV-positive patients (B).
